# Dynamic response and parameter identification of an impact oscillator with a one-sided elastic constraint

**DOI:** 10.1007/s11071-026-12946-2

**Published:** 2026-09-03

**Authors:** Bo Tian, Yang Liu, Julián Londoño Monsalve

**Affiliations:** 1 Exeter Small-Scale Robotics Laboratory, Engineering Department, University of Exeter, North Park Road, Exeter, EX4 4QF UK; 2 Engineering Department, University of Exeter, North Park Road, Exeter, EX4 4QF UK

**Keywords:** Nonlinear dynamics, Vibro-impact systems, System identification, Genetic algorithm optimisation, Backbone curve analysis, Stiffness estimation

## Abstract

Impact systems are common in engineering and exhibit inherently nonlinear and nonsmooth dynamics due to intermittent contact and elastic deformation. This study investigates the dynamic response and parameter identification of an impact oscillator with a one-sided elastic constraint. A custom experimental rig was developed to generate controlled vibro-impact responses, and a piecewise-smooth mathematical model was formulated to capture the unilateral contact dynamics. Two complementary parameter identification strategies, a genetic-algorithm-based optimisation and a backbone-curve fitting method derived from harmonic balance principles, were employed to estimate key system parameters, including impact stiffness, damping, and gap, using both displacement–frequency and time-domain data. The identified parameters agreed consistently with theoretical predictions across repeated experimental trials, demonstrating the repeatability of the proposed identification framework for the stable periodic responses observed under the forward stepped-sine excitation tests. Overall, the study provides an experimentally supported framework for identifying nonlinear characteristics of impact oscillators, with potential applications in structural dynamics, vibration mitigation, and biomedical diagnostics.

## Introduction

Impact systems arise in a wide range of engineering applications, including vibro-impact moling [1, 2], medical percussion diagnostics [3], impact dampers [4–6], energy harvesting systems [7, 8], and self-propelled capsule robots [9–12]. These systems exhibit complex nonlinear behaviours due to intermittent contact, clearance, and elastic deformation, all of which strongly influence performance, durability, and reliability. A common approach to studying such systems is to employ simplified oscillator models with impact constraints, which provide insight into bifurcations, nonlinear resonances, and energy transfer mechanisms. For instance, Nordmark et al. [13] analysed complete chattering in nonsmooth impact systems using discontinuity mappings, while Liu et al. [14] investigated multistability and bifurcations in a vibro-impact rig with two-sided constraints through combined modelling and experiments. Yin et al. [15, 16] further identified degenerate grazing bifurcation points and proposed strategies for suppressing grazing-induced instabilities. These studies collectively highlight the dominant role of impact-interface parameters in governing system responses.

Accurate identification of parameters such as impact stiffness, damping, and boundary compliance is therefore essential, as they directly influence vibration amplitudes, force transmission, and fatigue life. Reliable parameter identification enables more predictive simulations, supports structural safety assessments, and informs the design of vibration mitigation strategies. Numerous identification approaches for nonlinear systems have been proposed in recent years, including nonparametric methods [17–19], machine learning techniques [20–22], and subspace or Bayesian frameworks [23, 24]. For example, Zhao et al. [25] developed a physics-informed deep sparse regression network that integrates sparse regression with a Hybrid-LSTM3 architecture to identify nonlinear dynamics from noisy partial measurements. Liang et al. [26] proposed a polynomial nonlinear state-space model combined with multiple step inputs and an adaptive Nelder–Mead simplex algorithm to describe the nonlinear dynamics of an air turbine rocket engine. A physics-aware meta-optimisation method was also introduced to auto-tune unscented Kalman filters for robust identification under noisy or incomplete data [27]. These methods offer important advantages for different classes of nonlinear systems. For instance, machine-learning-based approaches can represent complex nonlinear input–output relationships, while Bayesian and filtering-based methods provide a systematic route for treating measurement noise and uncertainty. However, their application to vibro-impact systems may be limited by the need for large training datasets, probabilistic assumptions, or increased computational cost, and the resulting models may not always provide direct physical estimates of contact stiffness, damping, or impact gap.

Despite these advances, parameter identification in impact systems remains challenging. The inverse problem is intrinsically non-convex due to unilateral contact, piecewise-defined stiffness laws, and discontinuous restoring forces. Gradient-based optimisation methods, such as classical gradient descent [28–30] and quasi-Newton schemes [31–33], are generally unsuitable because they rely on smoothness assumptions and are highly sensitive to local minima. Although such methods are computationally efficient for smooth nonlinear systems, their performance can deteriorate when the objective function contains non-smooth transitions caused by intermittent contact. To overcome these limitations, a variety of global and metaheuristic algorithms have been explored, including stochastic search [34–36], evolutionary computation [37, 38], and nature-inspired optimisation [39–42]. For instance, Kao et al. [43] validated a modified particle swarm optimisation scheme for identifying Bouc–Wen hysteresis parameters, while Subudhi and Jena [44] demonstrated improved identification accuracy using an opposition-based differential evolution neural network. Among these methods, genetic algorithms (GAs) [45–47] have attracted significant interest due to their robustness in exploring high-dimensional parameter spaces, insensitivity to initial guesses, and successful application in structural identification problems.

Beyond optimisation algorithms, backbone curves [48, 49] have emerged as a powerful tool for identifying nonlinear parameters. By capturing the amplitude–frequency dependence of free or forced oscillations, backbone curves offer compact descriptors of effective stiffness and damping at varying oscillation levels. Londoño et al. [50, 51] employed zero-crossing techniques to extract backbone curves and damping ratios from resonant decay in lightly damped systems, whereas Ondra et al. [52] extended such methods to identify asymmetric restoring forces. More recently, Safari and Londoño Monsalve [53] proposed a cascaded optimisation framework for data-driven identification of asymmetric stiffness and damping nonlinearities. Backbone curves have also been used to validate identified parameters, ensuring, for example, that particle-filter-based estimates capture the underlying nonlinear stiffness and damping correctly [54]. Furthermore, Karaağaçlı and Özgüven [55] demonstrated that stepped-sine testing combined with the harmonic force surface approach can yield reliable amplitude-dependent nonlinear modal parameters for strongly nonlinear structures.

**Fig. 1:**
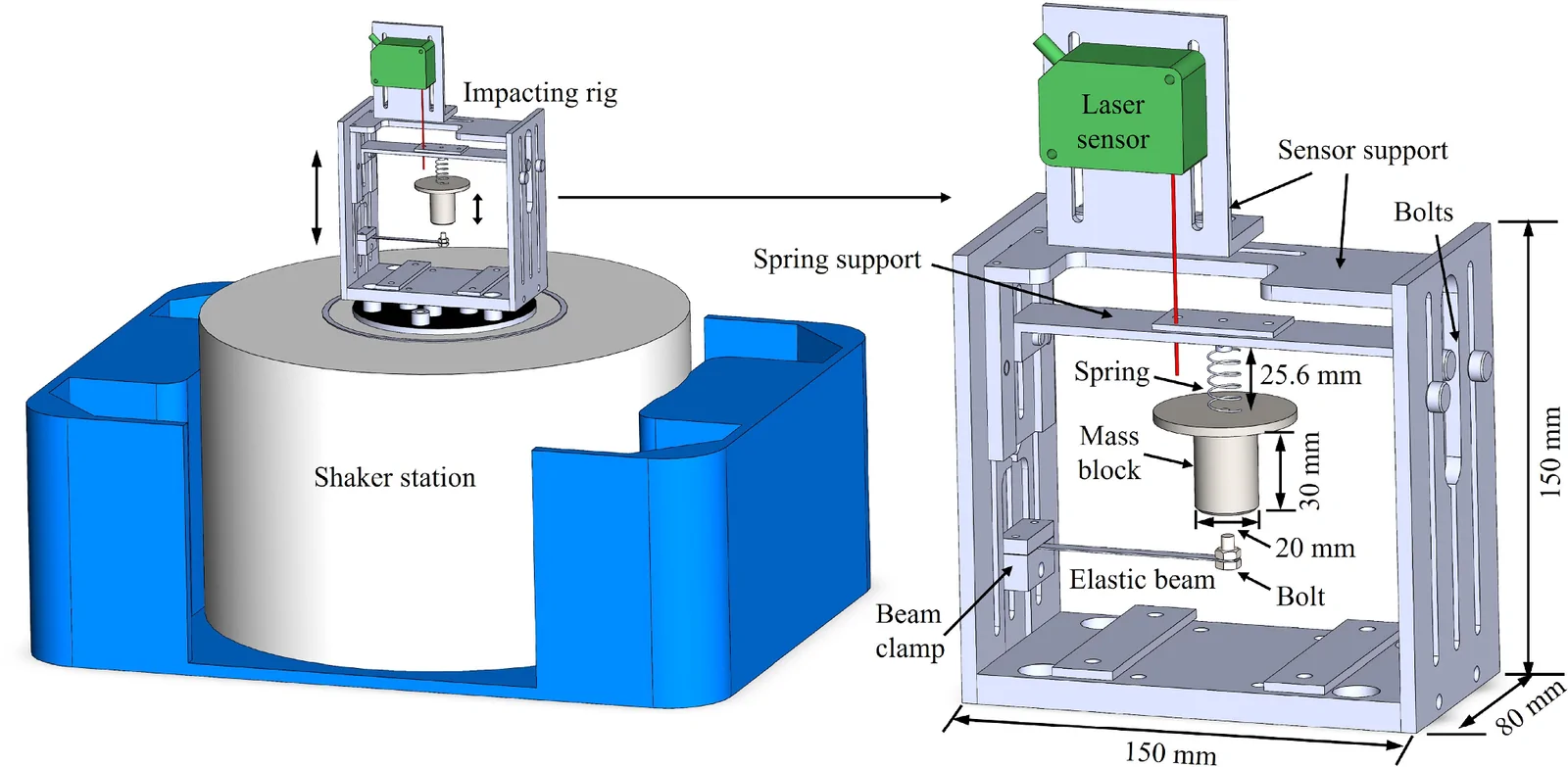
Schematic diagram of the forced-impact rig, where an impact oscillator (150 × 80 × 150 mm) is mounted on a shaker station. The rig includes a mass block connected to a helical spring (18.2 × 25.6 mm), fixed to an adjustable spring support. An elastic beam is secured using a repositionable beam clamp, allowing the impact gap to be adjusted. Impacts occur when the mass block strikes a bolt mounted on the beam. A laser displacement sensor is positioned vertically above the mass to capture its motion

In this paper, a harmonically excited mass interacting with an elastic beam is investigated as a representative experimental vibro-impact system. A purpose-built impact oscillator rig is developed, in which an electrodynamic shaker provides controlled sinusoidal base excitation and a laser displacement sensor measures the relative displacement response in both frequency-response and time-domain forms. Based on the experimental configuration, a physics-informed impact model with a one-sided elastic constraint is formulated, and the corresponding inverse problem is solved by minimising the discrepancy between simulated and measured responses. In addition, backbone curves extracted from the experimentally observed resonance features are used as complementary physical indicators for characterising the nonlinear response and estimating the equivalent contact stiffness.

The present work extends our recent study [56], in which the same class of impact oscillator was investigated using free-decay responses and an adaptive differential evolution algorithm, and where the capabilities and limitations of backbone curves extracted via a zero-crossing method were examined for stiffness identification. In contrast, the present study considers a harmonically driven configuration and uses forced steady-state responses obtained from stepped sine-sweep tests. This shift from free-decay responses to forced responses is significant because sine-sweep testing is a standard experimental procedure in vibration analysis, and the resulting frequency-response and time-domain data contain complementary information about the nonlinear and non-smooth dynamics of the system.

The main contribution of this study is twofold. First, it establishes an experimentally grounded procedure for identifying contact-related parameters of a one-sided vibro-impact oscillator from forced steady-state response data. In particular, displacement-frequency response curves and selected time-domain responses are used to estimate an effective parameter set, with emphasis on the equivalent contact stiffness and impact gap. Second, it examines the consistency and limitations of different response features for parameter identification by comparing frequency-response-based identification, time-domain identification, and backbone-curve-based stiffness estimation within the same experimental system. The influence of the impact gap and the repeatability of the identified contact stiffness under repeated trials are further analysed to support the interpretation of the identification results.

The remainder of this paper is organised as follows. Section 2 presents the design of the impact oscillator and its governing mathematical model. Section 3 describes the experimental setup and displacement measurement procedure. Section 4 provides a detailed analysis of the experimental results, including stiffness identification via both genetic algorithm (GA) based optimisation and backbone curve techniques. The influence of the experimental gap on the identification process and the repeatability of the estimated parameters are also examined. Finally, conclusions are summarised in Sect. 5.

## Design of an impact oscillator and establishment of the mathematical model under harmonic excitation

### Design of the impact oscillator

This section presents the design of an impact oscillator specifically developed to investigate dynamic behaviour under vibro-impact conditions and to support system identification using sine-sweep responses.

The impact rig consists of a rigid aluminium frame that supports all mechanical components, as illustrated in Fig. 1. At the core of the system is a vertically suspended mild steel mass block with a mass of 0.14 kg. The block is connected to a helical spring anchored to the upper frame, allowing the mass to move freely in the vertical direction. The spring support is mounted on a sliding rail and secured with bolts, enabling fine adjustments of the spring position and consequently the static height of the mass block.

Below the mass block, an elastic mild-steel beam is positioned to act as the impact obstacle. This beam is clamped to the frame using a beam clamp that can be repositioned along the same rail, allowing the impact gap between the mass and the beam to be adjusted. The effective length of the beam can also be modified by selecting clamps of different widths, offering further control over its stiffness.

The entire assembly is mounted on a VENZO 820 electrodynamic shaker, which provides controlled sinusoidal excitation over a broad frequency range through a DTC shaker control system. Impacts occur when the descending mass block strikes the elastic beam. To capture the system response, a laser displacement sensor is mounted vertically above the mass block on a dedicated sensor bracket. This non-contact sensor provides high-resolution displacement measurements and can be repositioned to ensure optimal alignment.

**Fig. 2:**
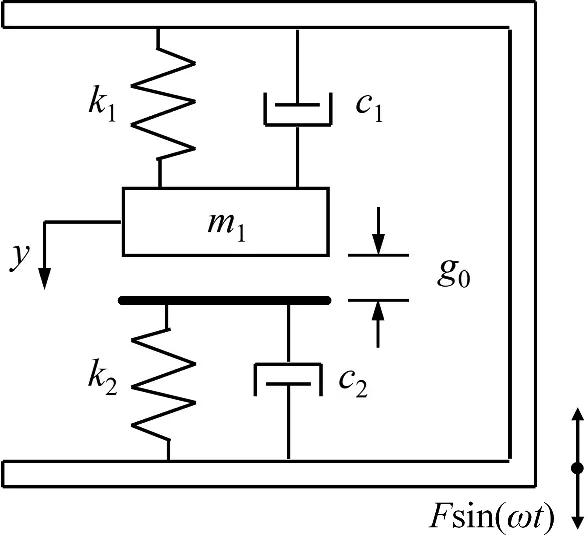
Mathematical model of the impact oscillator. Here, $m_1$ is the mass of the block, $k_1$ and $c_1$ denote the stiffness and damping of the primary linear spring-damper support, $k_2$ and $c_2$ represent the stiffness and damping of the one-sided elastic constraint, $g_0$ is the impact gap, and $F\sin (\omega t)$ is the applied excitation force

### Mathematical description of the impact oscillator

The system studied in this work is an impact oscillator experiencing intermittent soft impacts, as shown in Fig. 2. In mechanical systems, a soft impact refers to the collision between a body and an obstacle of negligible mass but non-negligible stiffness. The assumption of a negligible beam mass is justified because the beam mass is much smaller than that of the mass block. Moreover, the forced response examined here is dominated by the low-frequency motion of the mass block and the contact-induced stiffness variation, rather than by the high-frequency flexible vibration of the beam itself.

**Fig. 3:**
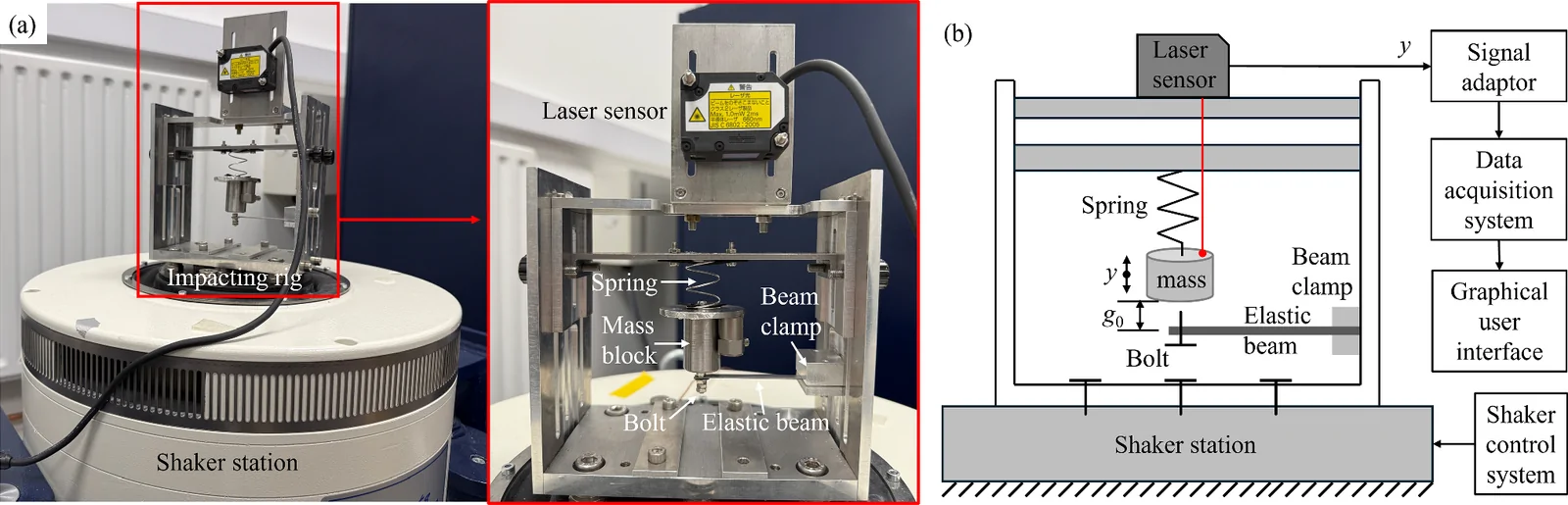
**a** Photograph and **b** schematic of the experimental apparatus. The impact rig is mounted on a shaker station controlled by a desktop computer, while displacement measurements from the laser sensor are transmitted to a laptop for acquisition and visualisation using a LabVIEW block diagram at a sampling rate of 1 kHz

In the present rig, the mass block $m_1$ is connected to the primary linear spring-damper support with stiffness $k_{1}$ and damping $c_{1}$. The elastic beam forms the one-sided constraint and is represented by the equivalent contact stiffness $k_{2}$ and damping $c_{2}$. The mass block is excited by a sinusoidal force and undergoes vertical motion. An impact occurs when the displacement of the block *y* reaches or exceeds the preset gap $g_0$. The discontinuity boundary is assumed to be neither motion-dependent nor time-varying.

The governing equation of motion is expressed as

1 $$\begin{aligned}&m_1 \ddot{y} + c_{1}\dot{y} + c_{2}\dot{y}\,H(y-g_0) \nonumber \\&\qquad + k_{1}y + k_{2}(y-g_0)\,H(y-g_0) = F \sin (\omega t), \end{aligned}$$

where H(·) is the Heaviside step function, and *F* and ω denote the forcing amplitude and angular frequency, respectively. The excitation amplitude from the shaker can be written as

2 $$\begin{aligned} F = m_2 a_0, \end{aligned}$$

where $m_2$ is the overall mass of the impact rig and $a_0$ is the manually set acceleration level in the shaker control system.

To ensure physical consistency, a non-negativity condition is imposed on the contact force:

$$ k_2(y-g_0) + c_2\dot{y} \ge 0. $$

Accordingly, the Heaviside function is defined as

3 $$\begin{aligned} H = {\left\{ \begin{array}{ll} 1, &  y-g_0 \ge 0 \ \text {and}\ k_2(y-g_0) + c_2\dot{y} \ge 0, \\ 0, &  \text {otherwise}. \end{array}\right. } \end{aligned}$$

## Experimental setup and procedure

This section describes the configuration of the experimental apparatus and the procedure used to acquire displacement measurements. The experimental setup is shown in Fig. 3. The impact rig was mounted on a shaker station connected to a cooling blower and a power amplifier. The shaker was operated using a desktop computer running dedicated control software capable of generating sinusoidal excitations with adjustable frequency, amplitude, and scan duration for each test. An accelerometer mounted on the shaker was used as the feedback signal for the controller. In each test, the desired acceleration level was prescribed in the software, and the measured acceleration from the accelerometer was fed back to the controller and displayed in the software interface. During the experiments, the displayed acceleration remained close to the prescribed value, confirming that the applied excitation level was consistent with the target value. Therefore, in this work, the target value is used as input to the numerical model. Under harmonic excitation, the entire rig oscillates vertically, causing the mass block to move up and down. It should be noted that the helical spring was mechanically connected to the mass block by pressing the lower end of the spring with a washer and fixing it through a bolt into a threaded hole machined in the mass block. Since the lower end of the helical spring is not perfectly flat, this connection may introduce a small geometrical asymmetry and lead to a slight inclination of the mass block. The small component visible on the mass block is an accelerometer mounted mainly as an auxiliary balancing component to reduce this inclination.

The present study uses displacement as the primary measured quantity because it is directly related to the impact gap and to the piecewise restoring-force law in the mathematical model. Compared with velocity or acceleration measurements, displacement signals are less sensitive to the high-frequency transients and sharp local distortions generated by impacts. In contrast, velocity and especially acceleration signals may contain pronounced impact-induced peaks and higher harmonic components, which can dominate the measured response if used directly in a time-domain identification procedure. Therefore, the identification framework in this work is formulated using displacement-based response features. Displacement measurements were obtained using a laser displacement sensor positioned above the mass block. Since the laser sensor was mounted on the vibrating frame, the recorded signal corresponds to the relative displacement of the mass block with respect to the frame. Therefore, all experimentally measured displacement responses reported in this study are interpreted as relative displacement responses. The sensor output was transmitted to a laptop via an NI USB-6210 data-acquisition unit. Data visualisation and recording were implemented using a LabVIEW block diagram, with a uniform sampling rate of 1 kHz applied throughout all experiments. To reduce noise, all displacement signals were smoothed using a Savitzky–Golay filter, which fits a second-order polynomial over a span of 11 data points. Since the sampling rate was 1 kHz, the filter window corresponds to 11 ms. The characteristic excitation frequencies considered in this study are mainly in the range of 10–17 Hz, corresponding to vibration periods of approximately 59 – 100 ms. Therefore, the filter window is substantially shorter than one vibration cycle and preserves the main temporal features required for extracting steady-state peak displacements and resonant frequencies. The filter may slightly smooth very sharp local variations associated with impact events. However, the parameter identification in this work is based on steady-state displacement amplitudes and frequency-response features rather than on the detailed high-frequency contact transient.

**Fig. 4:**
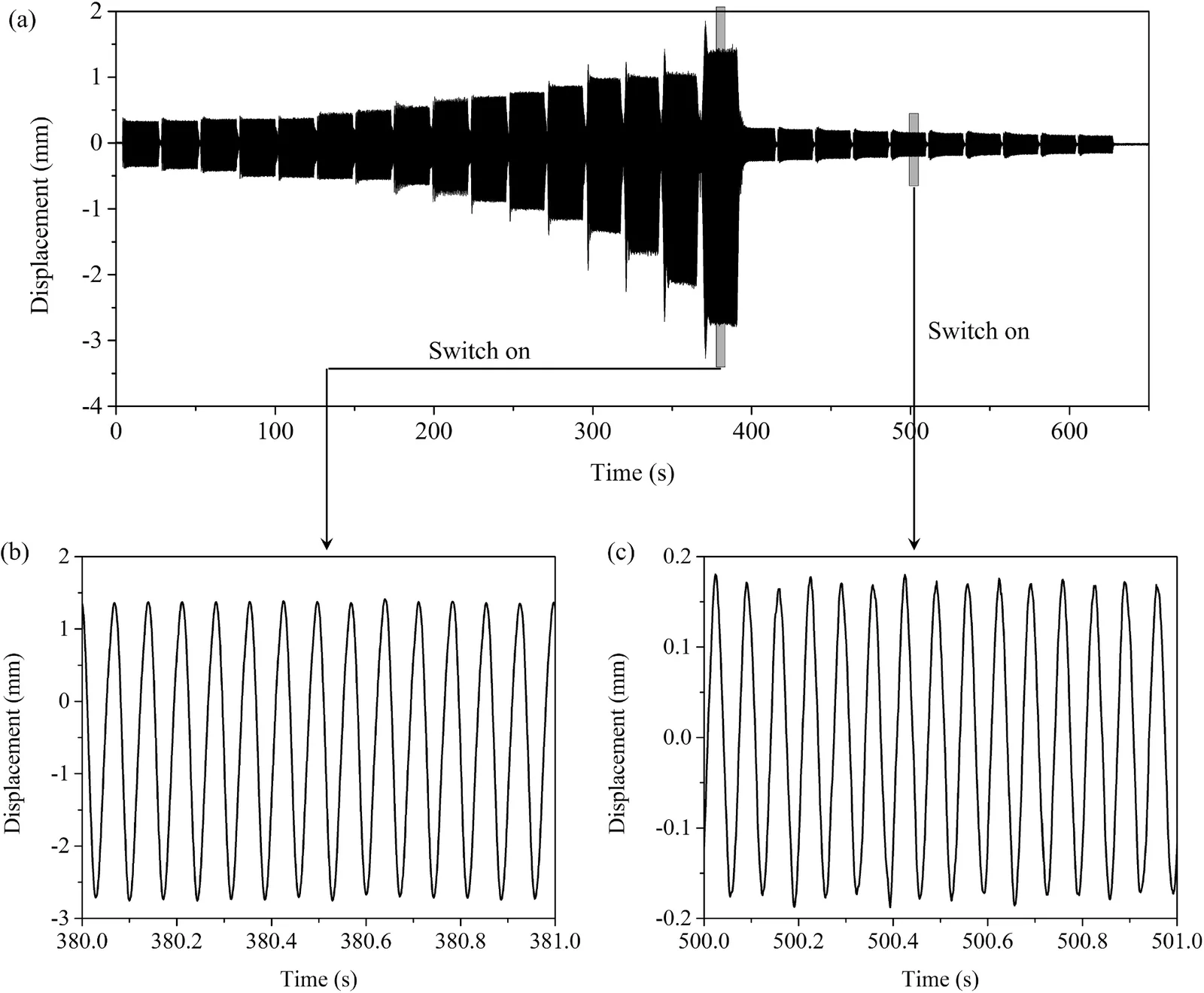
**a** Continuous time-displacement data of the impact rig subjected to a forward stepped-sine excitation test from 11 to 16 Hz in steps of 0.2 Hz. **b** and **c** Magnified sections of the response in **a** showing steady-state behaviour at selected frequencies

To illustrate the measurement procedure, a representative experiment was conducted using a frequency sweep from 11  to 16 Hz in increments of 0.2 Hz. Each frequency step was maintained for 20 s to ensure that the system reached a steady state. The resulting continuous displacement record is shown in Fig. 4. The label “switch on” marks a zoomed-in view of the sharp transition associated with the onset of contact.

As shown in Fig. 4a, the displacement amplitude first increases and then decreases as the excitation frequency rises, with a peak observed at 14 Hz. The corresponding steady-state response at 14 Hz is presented in Fig. 4b. At and below 14 Hz, impacts occur between the mass block and the elastic beam, evidenced by the asymmetric waveform, which means the maximum positive deflection and the maximum negative deflection are not symmetric with respect to the equilibrium position because of the one-sided elastic constraint. Above 14 Hz, the response becomes symmetric, as shown in Fig. 4c, indicating that no impacts take place in this frequency range.

In this work, several scenarios with different secondary stiffness values $k_2$ were studied by altering the effective length and thickness of the elastic beam. The effective length was adjusted by selecting clamps of different widths. The theoretical stiffness of the elastic beam was calculated using

4 $$\begin{aligned} k_t = \frac{3E}{l^3} \cdot \frac{b h^3}{12}, \end{aligned}$$

where $k_t$ is the theoretical stiffness, and *l*, *b*, *h*, and *E* denote the beam length, width, thickness, and modulus of elasticity, respectively. The beam parameters and their corresponding theoretical stiffness values are listed in Table 1.

It is worth noting that all experimental data in this study were acquired using a forward stepped-sine excitation protocol, in which the excitation frequency was increased in discrete stages. At each excitation frequency, the transient response was excluded, and the final 1 s of the measured signal was used to extract the steady-state response quantities. The identification procedure focuses on the impact stiffness parameter $k_2$ associated with the experimentally observed stable periodic response branch. In vibro-impact systems, the resonance region may be sensitive to excitation history, and upward and downward sweeps may follow different response branches. Therefore, the identified parameters are interpreted within the scope of the forward stepped-sine response branch observed in the present experiments. Full characterisation of hysteresis ranges, branch transitions, and coexisting response regimes will be addressed in future work using upward and downward sweeps or continuation-based experimental testing.

**Table 1:** Parameters of the three elastic beams used in the experiments

Case	*l* (mm)	*h* (mm)	*b* (mm)	*E* (MPa)	$k_t$ (N/m)
1	65	0.95	5	$1.75 \times 10^5$	682.9
2	57	0.95	5	$1.75 \times 10^5$	1012.7
3	45	0.95	5	$1.75 \times 10^5$	2058.1

Here, *l*, *b*, *h*, and *E* denote the length, width, thickness, and modulus of elasticity of the elastic beam, respectively, and $k_t$ is the corresponding theoretical stiffness

## Analysis of experimental results

In this section, two parameter identification methods are employed to determine the stiffness of the elastic beams based on the experimental data. The first method uses a GA, while the second relies on the backbone curves of the impact system. To quantify the accuracy of the identified stiffness parameter, the relative error $E_{k_2}$ between the identified stiffness $k_2$ and the theoretical beam stiffness $k_t$ is calculated by using $\vert k_2-k_t\vert /k_t \times 100\%$.

### Parameter identification using a genetic algorithm

A range of optimisation algorithms has been developed for parameter identification, including evolutionary algorithms [57], simulated annealing [58], and GAs [45]. In this work, a GA-based method is adopted to identify the parameters of the model defined in Eq. (1). The inverse problem considered here is non-convex and nonsmooth because of the unilateral contact condition, the piecewise stiffness law, and the discontinuous activation of the impact force. These features make gradient-based local optimisation methods less suitable, as the objective function may contain multiple local minima and may not be differentiable with respect to all parameters. The advantage of using a GA in the present framework is its population-based global search capability, its low sensitivity to the initial parameter guess, and its straightforward implementation with bounded design variables. These features are useful for simultaneously identifying the gap, damping coefficients, linear stiffness, and impact stiffness from displacement–frequency and time-domain response data. The proposed identification framework is not restricted to the GA itself; other derivative-free global optimisation algorithms could also be incorporated. The GA is used here to demonstrate the feasibility and repeatability of the proposed simulation–experiment matching framework.

For each candidate parameter set generated during the GA optimisation, Eq. (1) was solved in MATLAB using the stiff variable-step solver ode15s. The internal integration step size was adaptively determined by the solver, while the numerical displacement response was output at a fixed interval of 0.001 s to match the experimental sampling rate of 1 kHz. The relative and absolute tolerances were set to $10^{-6}$ and $10^{-8}$, respectively. The unilateral impact was handled using the Heaviside switching condition in Eq. (3), with the contact stiffness and damping terms activated only during valid contact.

The central idea is to reformulate the identification process as an optimisation problem, where the model parameters are adjusted so that the numerical solution of Eq. (1) reproduces the experimentally measured response under identical loading conditions. By minimising the mismatch between simulated and measured responses, the GA determines the parameter set that best characterises the physical system. In this study, the GA is used to estimate system parameters using two types of data: displacement–frequency response curves and time–displacement signals at the resonant frequency.

It should be noted that the present optimisation problem may not provide a unique global parameter set. Different combinations of mass, damping, stiffness, and gap may reproduce similar response features, particularly when only displacement response data are used. Therefore, the identified parameters are interpreted as effective parameters rather than as uniquely identifiable physical constants. In the present study, the main parameter of interest is the impact stiffness $k_2$, which is compared with the theoretical beam-stiffness estimate and assessed through repeated experiments. The remaining parameters, including $m_2$ and damping coefficients, may be more sensitive to the selected cost function because they can compensate for unmodelled effects such as excitation transmission, contact losses, local fixture compliance, and measurement perturbations.

#### Parameter identification based on the experimental displacement-frequency curves

In this study, the displacement-frequency curve is constructed by extracting the steady-state positive and negative peak displacements from the final 1 s of the steady response at each excitation frequency. Within this time window, the peak values were obtained cycle by cycle and averaged to represent the steady-state response at the corresponding frequency. This curve encapsulates critical dynamic characteristics of the system, including its resonant frequency. By aligning the numerically simulated curve with its experimental counterpart, the underlying system parameters can be effectively identified using a GA.

From a generic perspective, the parameter identification process can be reformulated as an optimisation problem, wherein the objective is to minimise a defined cost function as follows:

5 $$\begin{aligned} J_1(\boldsymbol{\theta }) = \sum _{i=1}^{N_f} \left( \text {MSE}_{\text {pos},i}(\boldsymbol{\theta }) + \text {MSE}_{\text {neg},i}(\boldsymbol{\theta }) \right) , \end{aligned}$$

where $J_1$ represents the cumulative error in the displacement frequency response curves between the simulated and experimental data; $N_f$ denotes the number of experimental conditions, corresponding to the different excitation levels discussed in this work; and $\text {MSE}_{\text {pos},i}$ and $\text {MSE}_{\text {neg},i}$ are respectively the mean square errors (MSE) for the maximum positive and negative displacements between the simulated and experimental data, which can be written as

6 $$\begin{aligned} \text {MSE}_{\text {pos},i}(\boldsymbol{\theta }) = \frac{1}{N} \sum _{j=1}^{N} \left( s_{\text {pos},j}(\boldsymbol{\theta }) - e_{\text {pos},j} \right) ^2, \end{aligned}$$

7 $$\begin{aligned} \text {MSE}_{\text {neg},i}(\boldsymbol{\theta }) = \frac{1}{N} \sum _{j=1}^{N} \left( s_{\text {neg},j}(\boldsymbol{\theta }) - e_{\text {neg},j} \right) ^2, \end{aligned}$$

where *N* is the number of measured points under each external excitation level; $s_{\text {pos},j}$ and $s_{\text {neg},j}$ are respectively the maximum positive and minimum negative displacements of the simulation under different frequencies; $e_{\text {pos},j}$ and $e_{\text {neg},j}$ are the corresponding maximum positive and minimum negative displacements of the experiment; and the unknown set of parameters denoted by $\boldsymbol{\theta }$ for the impact oscillator can be expressed as

8 $$\begin{aligned} \boldsymbol{\theta } = \left[ g_0; m_2; c_1; c_2; k_1; k_2 \right] . \end{aligned}$$

It is worth noting that the parameters $g_0$, $m_2$, $c_1$, $c_2$, $k_1$ and $k_2$ are treated as global parameters in the optimisation process, and remain constant across all excitation levels, to ensure the robustness of the identified parameters. Ultimately, the optimal set of parameters $\boldsymbol{\theta }$ can be determined by solving the following optimisation problem,

9 $$\begin{aligned} \begin{aligned}&{\min } \ \ f=J_1(\boldsymbol{\theta }), \\&s.t. \ \ \ 0 \le g_0 \le 3 \ \text {mm}, \ 0.8 \ \text {kg}\le m_2 \le 1.5 \ \text {kg}, \\&\ 0 \le c_1 \le 1 \ \text {Ns/m}, \\&\ \ \ \ \ \ \ \ \ 0 \le c_2 \le 6\ \text {Ns/m}, \ 500\ \text {N/m} \le k_1 \le 1000\ \text {N/m}, \\&\ 0 \le k_2 \le 5000\ \text {N/m}, \end{aligned} \end{aligned}$$

where the search range for the stiffness parameter $k_2$ is determined based on the theoretical values $k_t$ of elastic beam in Table 1. A broad range is selected for the damping coefficient to account for its potential variability. The range for the gap $g_0$ is defined according to the experimental setup. The mass $m_2$ is estimated based on the measured weight of the whole rig, approximately 1.2 kg; Finally the range for $k_1$ is chosen based on the manufacturer’s specification of the spring, which is 800 N/m.

As for other algorithm settings, the population size was set to 100, the maximum number of generations was set to 200, the maximum number of stall generations was set to 20, and the function tolerance was set to $10^{-6}$. The crossover fraction was set to 0.8. The adaptive feasible mutation operator was used to generate mutation children while preserving the prescribed parameter bounds. The initial population was randomly generated within the specified parameter bounds. The same settings were used in Section 4.1.2.

**Fig. 5:**
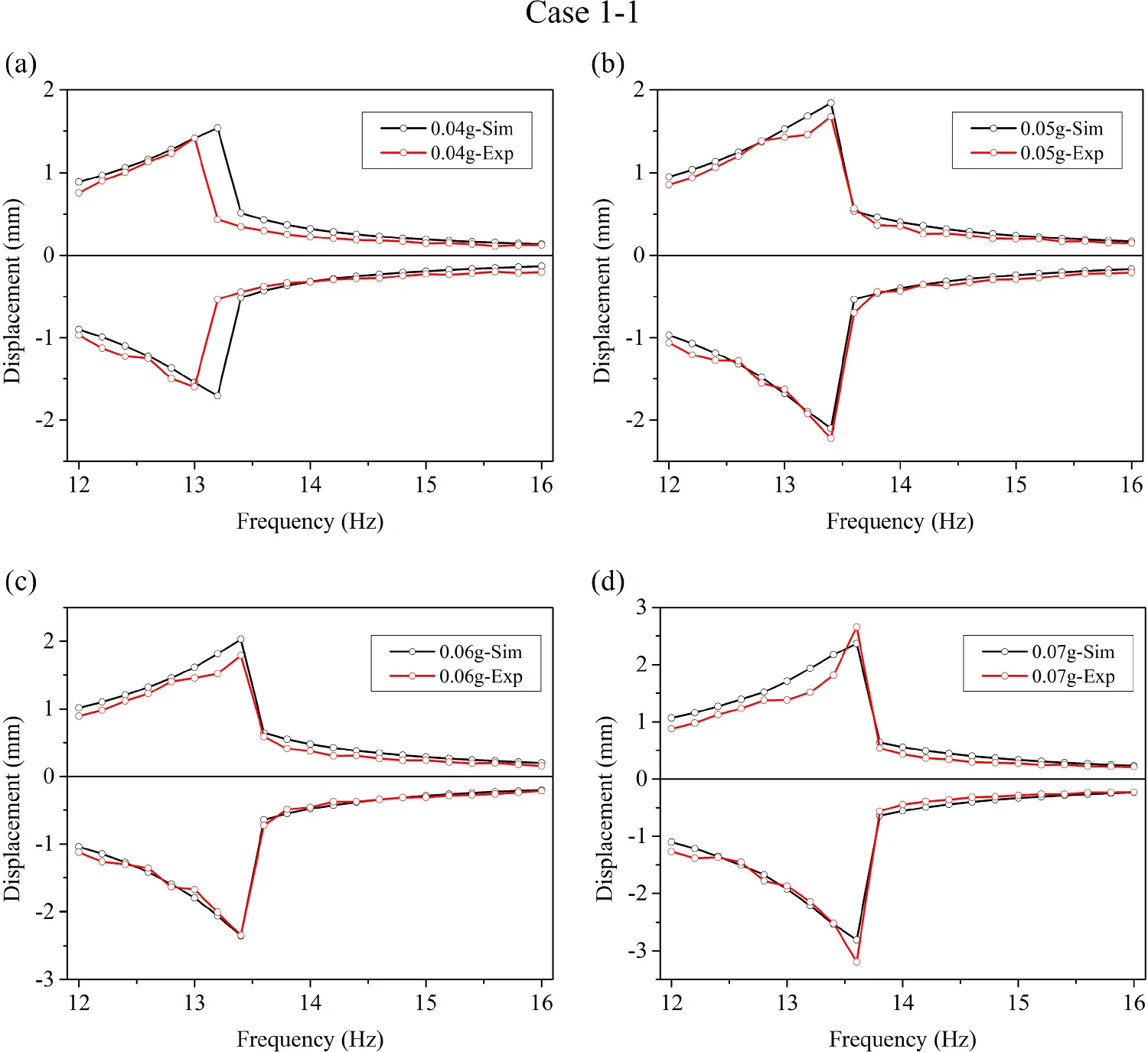
Comparison of the simulated (black solid curves with open-circle markers) and experimental (red solid curves with open-circle markers) positive- and negative-peak displacement–frequency response curves for Case 1-1, with a nominal experimental gap of $g_0 = 0.70$ mm, at four base-excitation acceleration levels: **a** 0.04 g, **b** 0.05 g, **c** 0.06 g and **d** 0.07 g

In the experimental investigations, both the external excitation acceleration, and hence the equivalent excitation-force amplitude, and excitation frequency were systematically varied based on the specific test case. For Cases 1 and 2, the excitation acceleration was set between 0.04 g and 0.07 g, with increments of 0.01 g, while the frequency ranged from 12 to 16 Hz in steps of 0.2 Hz. For Case 3, the excitation acceleration spanned from 0.06 to 0.12 g in 0.02 g increments, and the frequency ranged from 12 to 17 Hz, also in 0.2 Hz steps, where g denotes the standard acceleration due to gravity. The excitation force was applied through an electrodynamic shaker to base-excite the impact oscillator. The resulting time–displacement responses under each excitation condition were recorded using a high-precision laser displacement sensor. From the steady-state portions of these responses, the peak positive and negative steady-state displacements were extracted from the response data, thereby constructing the basis of displacement–frequency response curves.

**Fig. 6:**
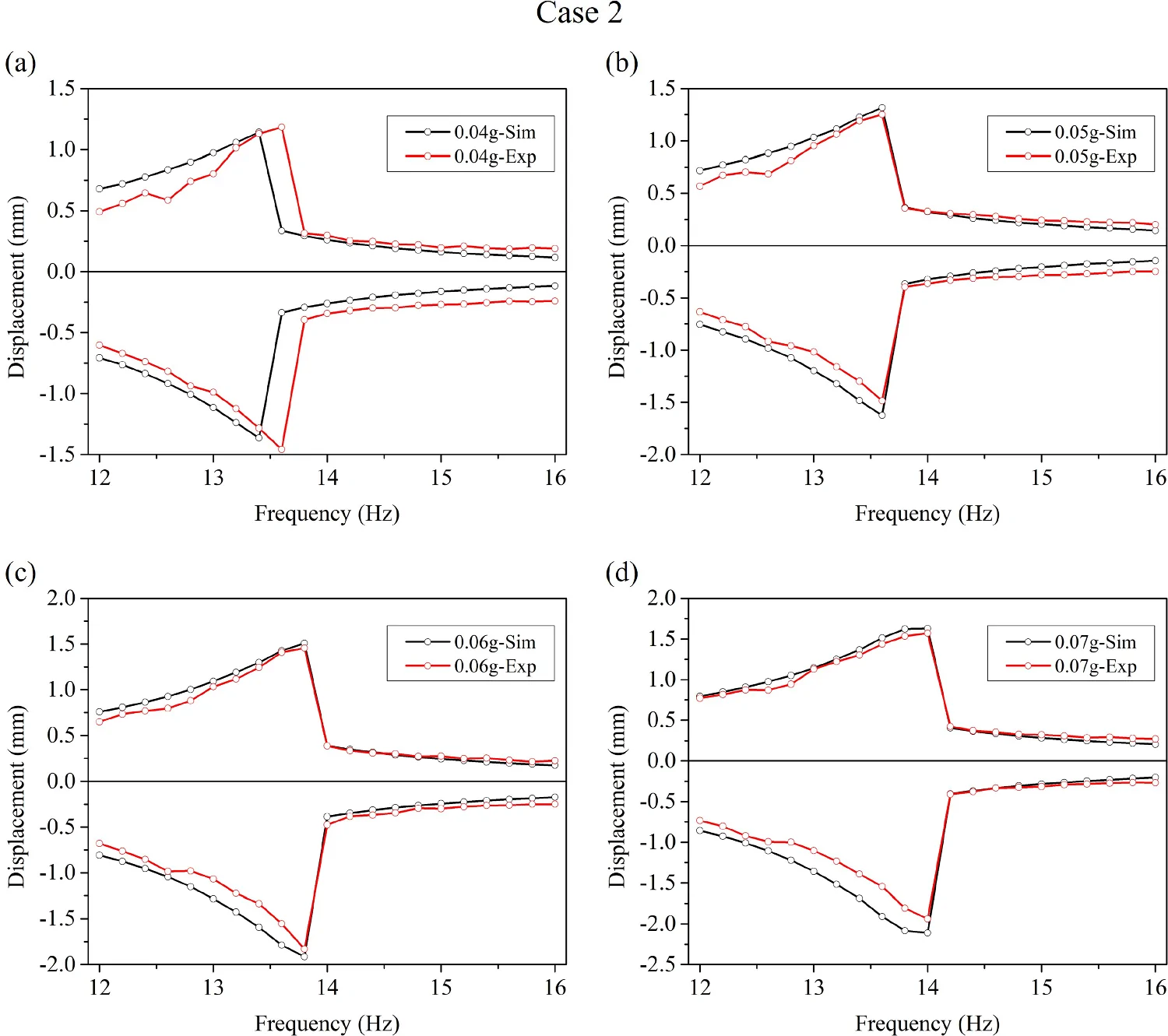
Comparison of the simulated (black solid curves with open-circle markers) and experimental (red solid curves with open-circle markers) positive- and negative-peak displacement–frequency response curves for Case 2, with a nominal experimental gap of $g_0 = 0.50$ mm, at four base-excitation acceleration levels: **a** 0.04 g, **b** 0.05 g, **c** 0.06 g and **d** 0.07 g

**Fig. 7:**
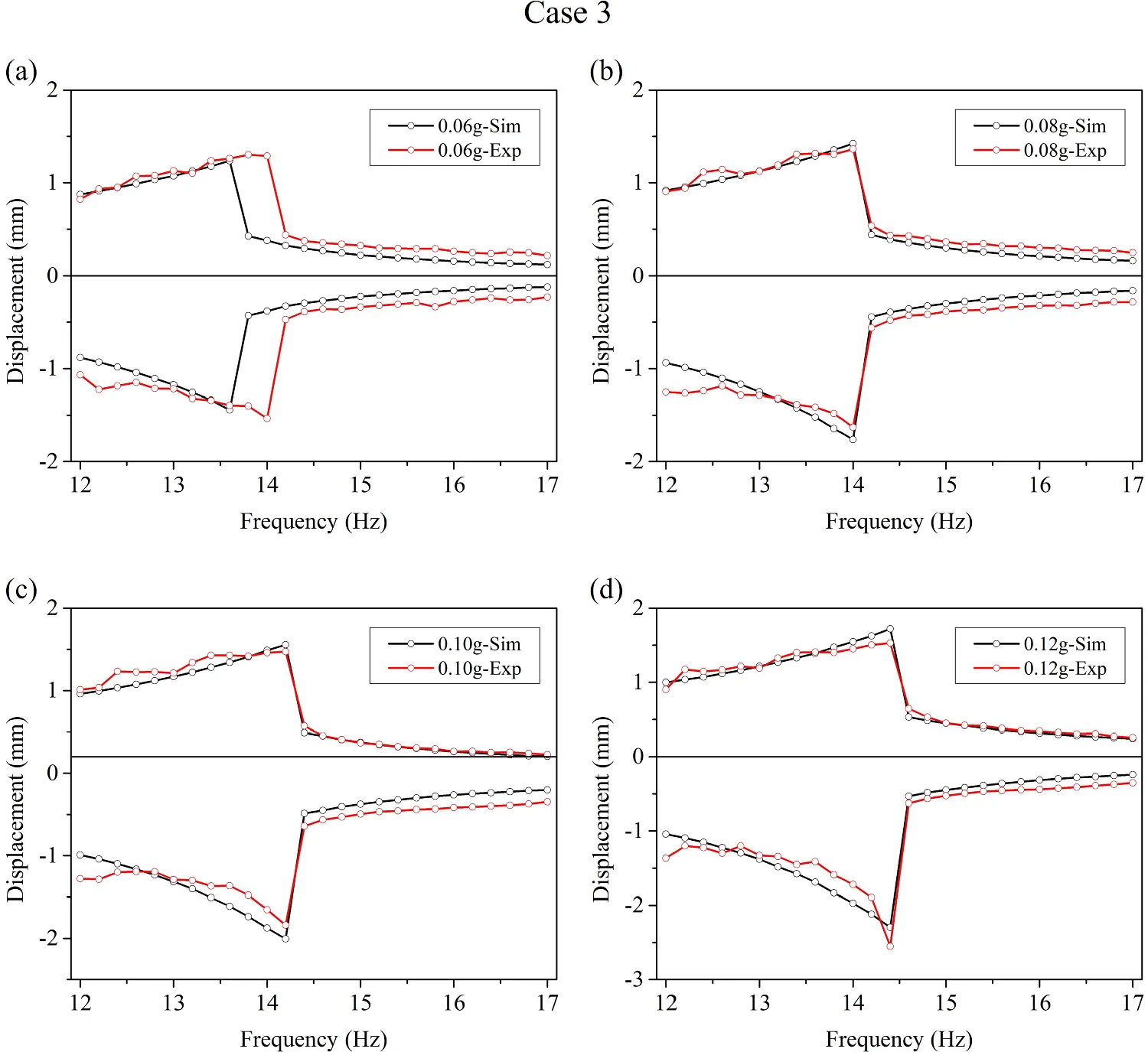
Comparison of the simulated (black solid curves with open-circle markers) and experimental (red solid curves with open-circle markers) positive- and negative-peak displacement–frequency response curves for Case 3, with a nominal experimental gap of $g_0 = 0.80$ mm, at four base-excitation acceleration levels: **a** 0.06 g, **b** 0.08 g, **c** 0.10 g and **d** 0.12 g

The displacement-frequency response curves under various excitation levels are shown in Figs. 5, 6 and 7, where the experimental results are depicted by red solid lines with open circular markers. These experimental curves clearly illustrate the system’s resonant behaviour, with the resonance frequency identified at the peak displacement in each case. As the excitation level increases from 0.04 to 0.07 g for Cases 1 and 2, 0.06 g to 0.12 g for Case 3, a consistent upward shift in the resonance frequency can be observed. This trend suggests a nonlinear stiffening effect within the system, where higher excitation amplitudes lead to increased dynamic stiffness, thereby shifting the natural frequency toward higher values.

Once all the experimental displacement frequency response curves were compiled, a GA was employed to identify the optimal parameter set $\boldsymbol{\theta }$ by solving Eq. (9). The identification results are detailed in Table 2. As indicated in the table, the identified values for parameters $k_2$ across three cases, which are 700.89 $\text {N/m}$, 1051.1983 $\text {N/m}$ and 2176.6722 $\text {N/m}$, are close to the corresponding theoretical stiffness values $k_t$, with relative errors of 2.63%, 3.80%, and 5.76%, respectively. These error levels indicate that the GA-based identification approach provides reasonable estimates of the stiffness parameter for the tested cases. Regarding the gap parameter $g_0$, the identified values for the three cases are 0.73162 mm, 0.49709 mm, and 0.77438 mm, respectively. These values exhibit good agreement with the experimentally configured gap settings of 0.70 mm, 0.50 mm, and 0.80 mm. Moreover, the relative errors between the identified values of the soft spring stiffness parameter $k_1$ and its theoretical value of 800 N/m are all within 6.5% across the three cases. This consistency further supports the physical plausibility of the identified parameter set. The observed discrepancies in the identified mass parameter $m_2$ may be attributed partly to small variations in the mass of the bolts and nuts used during beam fixation. In addition, the relatively thick cable connected to the laser displacement sensor may introduce small mechanical perturbations during shaker operation. Since the cable vibrates together with the experimental rig, it may slightly affect the sensor support, the alignment of the laser spot, or the measured displacement signal. These effects are not explicitly represented in the single-degree-of-freedom model and may therefore be partially absorbed into the identified effective mass parameter.

Subsequently, the mathematical model described by Eq. (1) was simulated using the previously identified parameters. The resulting displacement-frequency response curves, obtained from these simulations, are presented as black solid lines with open circular markers in Figs. 5, 6 and 7. By comparing the simulated results with experimental displacement-frequency curves, it can be seen in Fig. 5 that the identified model reproduces the main amplitude trend and peak-response frequency shift, particularly at higher excitation levels. A minor deviation in resonant frequency is observable at the lower excitation level. For Case 2 in Fig. 6, the simulation continues to align with experimental resonant frequencies. However, a more noticeable discrepancy in displacement amplitude, particularly on the negative displacement branch, is observed. For Case 3 in Fig. 7, the model captures the overall trend of the peak-response frequency, although local amplitude differences remain near resonance.

It should be noted that there are several potential sources of error that may contribute to the discrepancies observed between the experimental and simulated results. First, measurement noise inherent in the displacement sensing system may introduce minor fluctuations in the recorded response, particularly near resonance where signal sensitivity is high. Second, slight misalignment or tilting of the attached mass block during installation could lead to small lateral oscillations during vibration, resulting in deviations in the measured axial displacement that are not accounted for in the idealized model. Third, the frequency step size of 0.2 Hz used in the sine sweep procedure may be relatively large, potentially limiting the resolution with which the exact resonant frequency is captured. The resonance region of vibro-impact systems is also sensitive to excitation history. Coexisting stable periodic regimes, branch jumps, or regime transitions may occur depending on the initial condition and the frequency-stepping process. The comparisons in Figs. 5, 6 and 7 are therefore made with respect to the stable periodic response branch observed in the forward stepped-sine experiments. Within this scope, the identified $k_2$ provides a representative stiffness parameter for the experimentally realised periodic response regime. Finally, damping in the experimental system may be nonlinear and velocity dependent, whereas the model uses linear viscous damping, which may also contribute to amplitude and phase discrepancies.

**Fig. 8:**
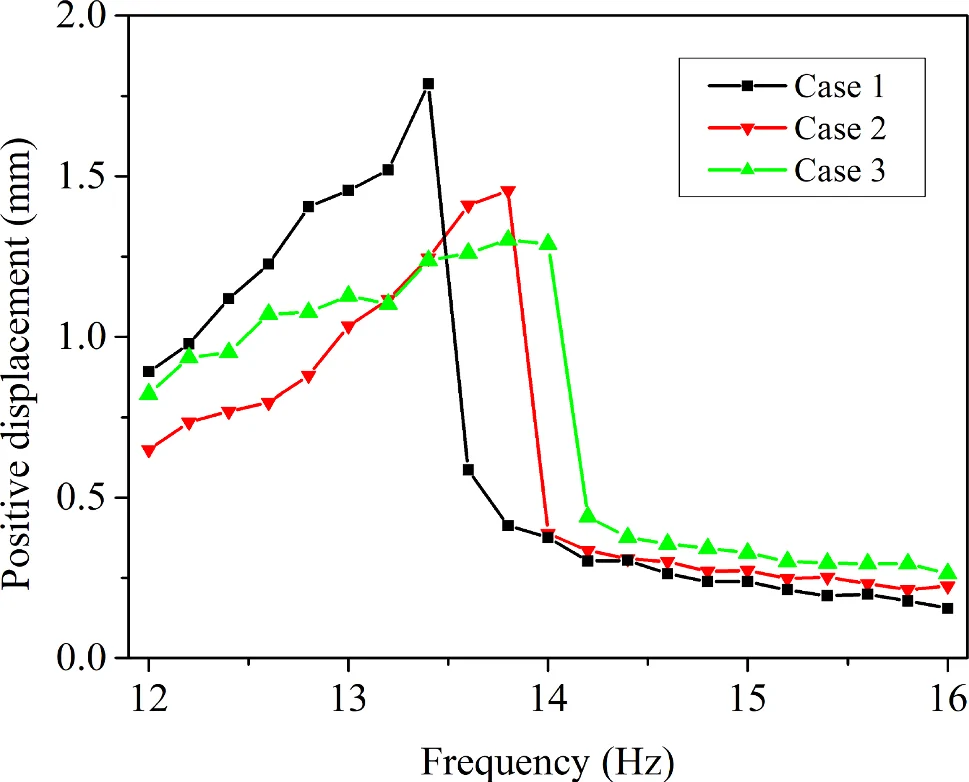
Comparison of the experimental displacement frequency response curves under the same external excitation acceleration (0.06 g) for three cases (Case 1 in black, Case 2 in red and Case 3 in green), where the higher stiffness of the elastic beam leads to a higher resonant frequency

By comparing the positive displacement-frequency curves under a fixed external excitation acceleration of 0.06 g for three cases, which are presented in black, red and green curves, respectively, it is evident from Fig. 8 that an increase in structural stiffness leads to a corresponding increase in resonant frequency.

#### Parameter identification based on the experimental time-displacement data

Furthermore, the system’s parameters are also identified based on the time-domain signals at the resonant frequencies. By aligning the numerically simulated curve with the corresponding experimental time-history data, the system’s parameters can be effectively estimated through the GA. The objective of this optimisation problem is formulated as follows:

10 $$\begin{aligned} J_2(\boldsymbol{\theta }) = \sum _{i=1}^{N} \sum _{j=1}^{T_i} \left( \hat{y}_i(t_j; \boldsymbol{\theta }) - y_i(t_j) \right) ^2, \end{aligned}$$

where $\boldsymbol{\theta }$ is the unknown set of parameters as expressed in Eq. (8). *N* denotes the total number of experimental datasets used in the optimisation, which is 4 in this study. Each dataset corresponds to a different excitation condition (e.g., amplitude and frequency). $T_i$ is the number of time samples in the *i*-th experimental dataset. $y_i(t_j)$ represents the experimentally measured displacement at time $t_j$ in the *i*-th dataset. $\hat{y}_i(t_j; \boldsymbol{\theta })$ denotes the simulated displacement at time $t_j$ in the *i*-th dataset, obtained from the numerical model using parameter vector $\boldsymbol{\theta }$.

**Table 2:** The results of parameter identification from the GA based on the displacement-frequency curves

Case	$g_0$ (mm)	$m_2$ (kg)	$c_1$ (Ns/m)	$c_2$ (Ns/m)	$k_1$ (N/m)	$k_2$ (N/m)	$k_t$ (N/m)	$E_{k_2}$
1	0.73162	1.3834	0.3777	0.89883	847.6022	700.89	682.9	2.63%
2	0.49709	1.2446	0.063365	2.6522	818.2683	1051.1983	1012.7	3.80%
3	0.77438	1.0347	0.047585	3.917	849.5509	2176.6722	2058.1	5.76%

The optimal parameter set $\boldsymbol{\theta }$ can be determined by solving the optimisation problem in Eq. (11) using a GA.

11 $$\begin{aligned} \begin{aligned}&{\min } \ \ f=J_2(\boldsymbol{\theta }), \\&s.t. \ \ \ 0 \le g_0 \le 3 \ \text {mm}, \ 0.8 \ \text {kg}\le m_2 \le 1.5 \ \text {kg}, \\&\ 0 \le c_1 \le 1 \ \text {Ns/m}, \\&\ \ \ \ \ \ \ \ \ 0 \le c_2 \le 6\ \text {Ns/m}, \ 500\ \text {N/m} \le k_1 \le 1000\ \text {N/m}, \\&\ 0 \le k_2 \le 5000\ \text {N/m}, \end{aligned} \end{aligned}$$

**Fig. 9:**
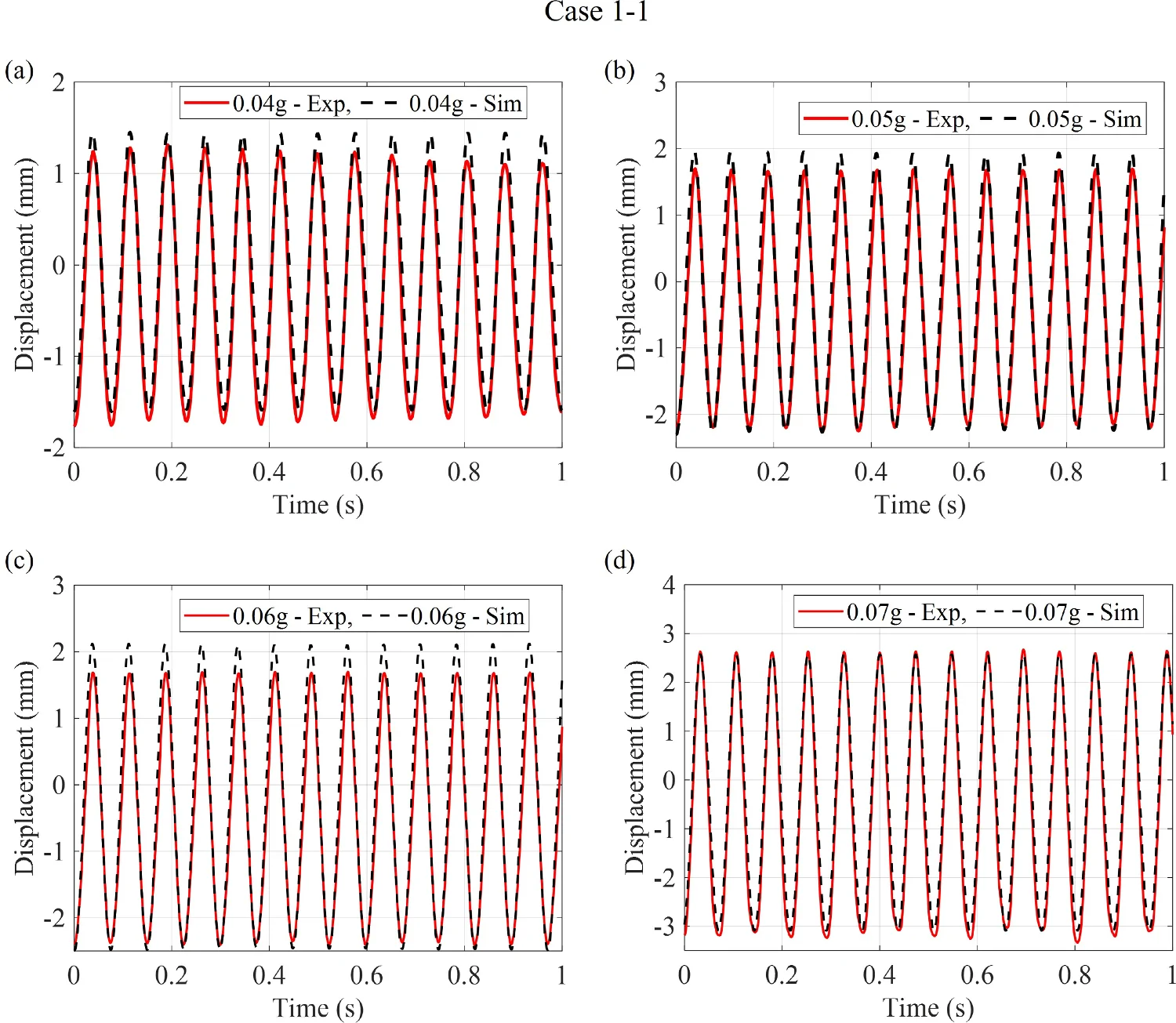
Comparison of the simulated (black dashed curves) and experimental (red solid curves) steady-state time–displacement responses at the experimentally determined resonant frequencies for Case 1-1, with a nominal experimental gap of $g_0 = 0.70$ mm, at four base-excitation acceleration levels: **a** 0.04 g, **b** 0.05 g, **c** 0.06 g and **d** 0.07 g

**Fig. 10:**
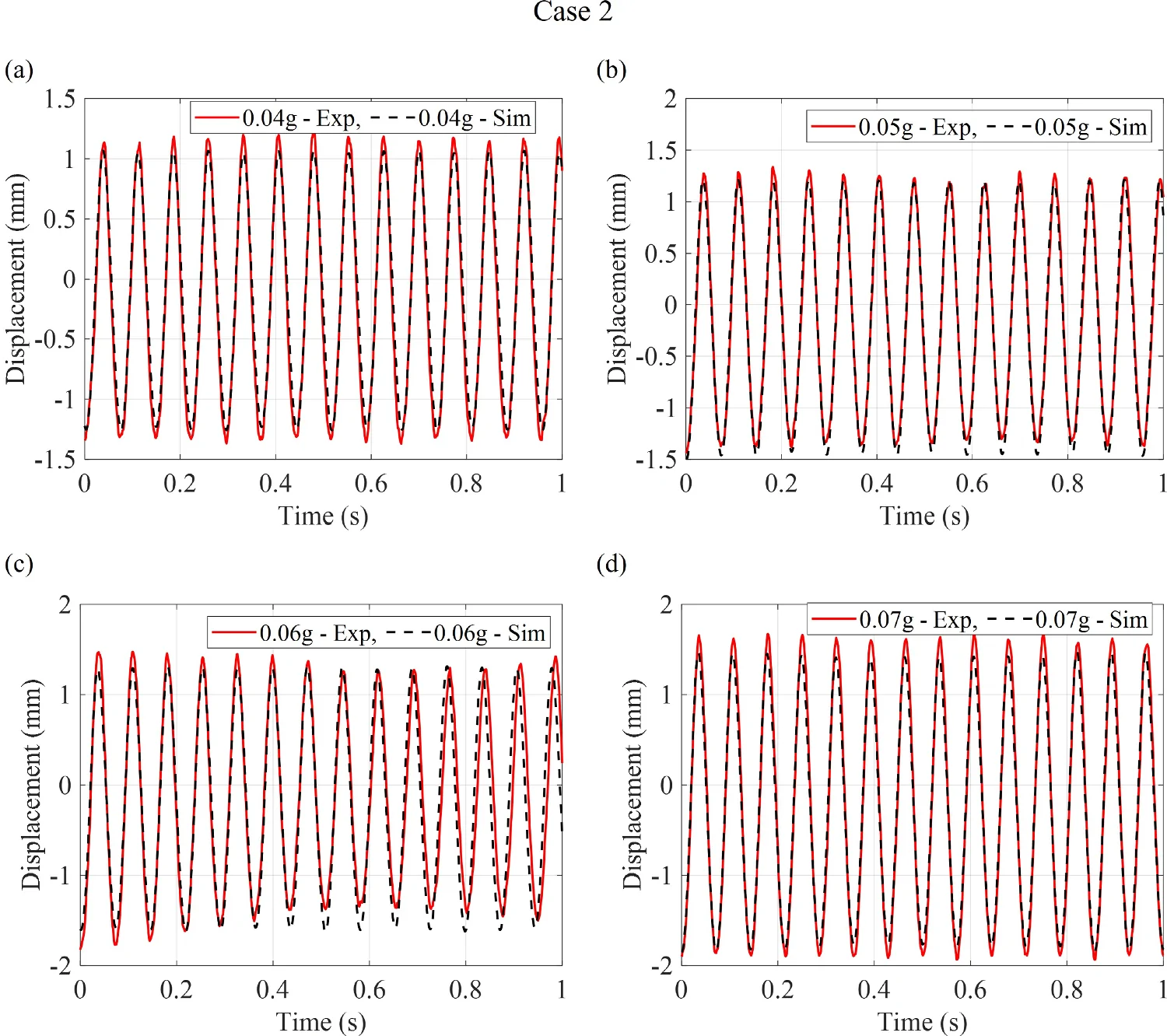
Comparison of the simulated (black dashed curves) and experimental (red solid curves) steady-state time–displacement responses at the experimentally determined resonant frequencies for Case 2, with a nominal experimental gap of $g_0 = 0.50$ mm, at four base-excitation acceleration levels: a 0.04 g, b 0.05 g, c 0.06 g and d 0.07 g

**Fig. 11:**
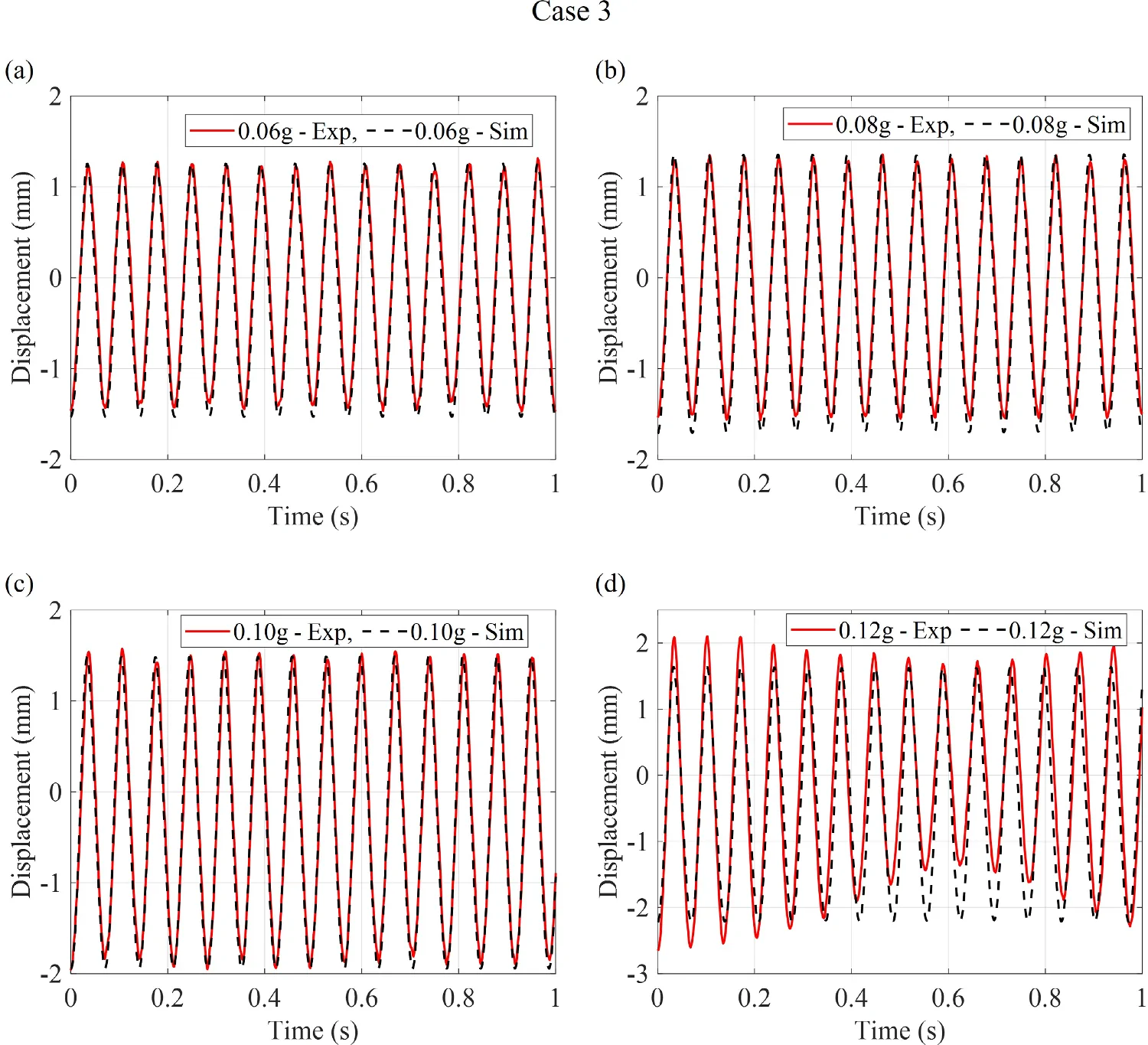
Comparison of the simulated (black dashed curves) and experimental (red solid curves) steady-state time–displacement responses at the experimentally determined resonant frequencies for Case 3, with a nominal experimental gap of $g_0 = 0.80$ mm, at four base-excitation acceleration levels: a 0.06 g, b 0.08 g, c 0.10 g and d 0.12 g

For all experiments, a one-second segment of steady-state time history data was selected for the optimisation process. Transient responses immediately after switching to a new excitation frequency were excluded. The selected window was checked to ensure that the response amplitude no longer showed a visible growth or decay trend. With the sampling frequency of 1 kHz, each selected segment contained 1000 time samples. The same steady-state time window was used for the corresponding numerical response when evaluating the time-domain mismatch. The results of the parameter identification are presented in Table 3. As can be seen from the table, the three identified values of $k_2$ closely match the theoretical values, as well as the values identified from the displacement–frequency curves. Subsequently, the impact model was simulated using the identified parameters and corresponding excitation inputs to compare the simulated time histories with the experimental results, as shown in Figs. 9, 10 and 11. In all three cases, the identified time histories are denoted by black dashed lines, while the experimental data are indicated by red solid lines. For Case 1 (Fig. 9), the identified time histories reproduce the dominant periodic response and the main impact-related features of the experimental data, although local amplitude differences are still visible. For Cases 2 and 3 (Figs. 10 and  11), discrepancies are observed at excitation levels of 0.06 g and 0.12 g, respectively. These differences are mainly reflected in the local amplitude and waveform near the impact events. A possible reason is that the experimental system does not behave as an ideal single-degree-of-freedom oscillator during contact. In particular, a slight inclination of the mass block or imperfect vertical alignment between the mass block, bolt, and elastic beam may introduce weak lateral motion. This can alter the effective contact location, contact duration, and impact timing, which are not represented in the simplified bilinear model. Overall, the comparisons shown in Figs. 9, 10 and 11 indicate that the identified parameters listed in Table 3 are effective.

**Table 3:** The results of parameter identification from the GA based on the time history data

Case	$g_0$ (mm)	$m_2$ (kg)	$c_1$ (Ns/m)	$c_2$ (Ns/m)	$k_1$ (N/m)	$k_2$ (N/m)	$k_t$ (N/m)	$E_{k_2}$
1	0.70	1.3825	0.1082	1.6673	845.67	721.97	682.9	5.72%
2	0.47	1.4878	0.1134	3.7891	821.37	950.83	1012.7	6.11%
3	0.75	1.4952	0.2472	4.9969	849.84	2051.41	2058.1	0.33%

### Parameter identification using backbone curves

In addition to the GA, a backbone-curve-based formulation is employed as a complementary method for estimating the nonlinear stiffness characteristics of the impact oscillator. In the present study, the backbone-related points are extracted from the maximum positive steady-state responses observed in the forward stepped-sine sweeps at different excitation levels. The stepped-sine tests were conducted over a sufficiently wide frequency range to capture the observed resonance region for each experimental case. The extracted peak-response points are therefore used to describe the amplitude-dependent resonance trend observed from the forward-sweep measurements. It should be noted that these experimentally extracted points do not represent a complete nonlinear continuation of all possible response branches. In particular, possible overhanging branches, hysteresis regions, or unstable branches cannot be fully determined from forward sweeps alone. The backbone-curve-based procedure is therefore used here as an approximate stiffness-estimation method and is interpreted together with the GA-based identification, theoretical stiffness values, and repeated experimental trials.

The harmonic balance method [59, 60] is widely used to derive the expressions for the effective stiffness of nonlinear systems. This method, which emulates the behaviour of a spectrum analyser, assumes that the response to a sinusoidal excitation remains a sinusoid at the same frequency. Accordingly, for the impact system under sinusoidal excitation, a trial solution of the form $y = Y\text {sin}(\omega t)$ is assumed and substituted into the equation of motion given in Eq. (1). This corresponds to a single-term harmonic balance approximation, in which only the fundamental harmonic component of the displacement response is retained. Higher harmonic components generated by the nonsmooth impact process are not included in this simplified analytical treatment. This assumption is applied to the dominant displacement component of the response. If velocity or acceleration measurements were used for the backbone-curve-based estimation, the response amplitude should not be taken directly from the raw peak velocity or acceleration. Impact events introduce higher harmonics and sharp transient components, which are much more pronounced in velocity and acceleration than in displacement. Directly using these distorted peak values would lead to an overestimation of the equivalent response amplitude and would reduce the accuracy of the stiffness estimate. A more appropriate procedure would be to extract the fundamental harmonic component at the excitation frequency and convert it to an equivalent displacement amplitude before applying the effective-stiffness formulation. Since the response of this system is asymmetric and the primary aim of this study is to identify stiffness $k_2$, only the positive backbone curve is considered. Accordingly, by focusing on the positive peak displacement $y_\text {pos}$ at resonant frequency, the resulting expression for the effective stiffness of the impact system is given in Eq. (12). A detailed derivation can be found in Appendix A.

12 $$\begin{aligned} k_{\text {eq}}&= k_1 + \frac{k_2}{2\pi } \left[ \pi - 2 \theta _c + \sin (2\theta _c) - \frac{4 g_0}{y_\text {pos}} \cos \theta _c \right] , \end{aligned}$$

where $\theta _c$ is the phase angle at which the displacement *y* first reaches the gap $g_0$, and it is defined as

13 $$\begin{aligned} \theta _c = \sin ^{-1}\left( \frac{g_0}{y_\text {pos}}\right) . \end{aligned}$$

By substituting Eq.(13) into Eq.(12), the effective stiffness can be defined as

14 $$\begin{aligned} k_{\text {eq}} =\  &k_1 + \frac{k_2}{2\pi } \bigg \{ \left( \pi - 2\sin ^{-1} \left( \frac{g_0}{y_\text {pos}} \right) \right) \nonumber \\&+ \sin \left[ 2\sin ^{-1} \left( \frac{g_0}{y_\text {pos}} \right) \right] \nonumber \\&- \frac{4g_0}{y_\text {pos}} \cos \left[ \sin ^{-1} \left( \frac{g_0}{y_\text {pos}} \right) \right] \bigg \}. \end{aligned}$$

**Fig. 12:**
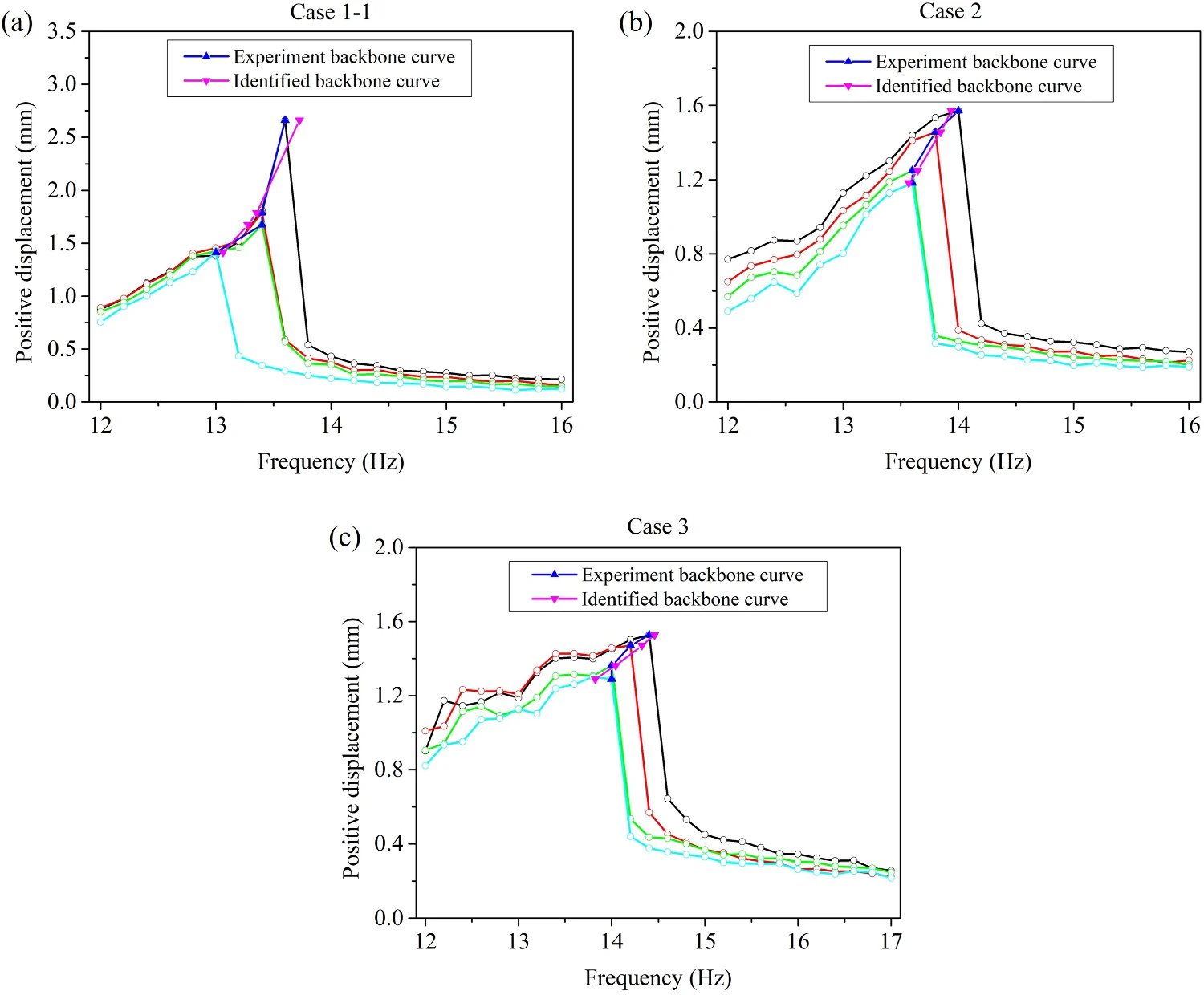
Experimental backbone curves (blue upward-triangle markers) and identified backbone curves (pink downward-triangle markers) extracted from displacement–frequency responses at different base-excitation acceleration levels for **a** Case 1-1, with $g_{0}$ = 0.70 mm, **b** Case 2, and **c** Case 3

Once the expression for the effective stiffness is derived, the stiffness $k_2$ can be determined by fitting it to the experimental backbone curve. The experimental backbone curves for the three cases are constructed by connecting the peak displacements at the resonant frequencies corresponding to various excitation levels. Since the impact oscillator is a nonsmooth, piecewise-linear system, and the primary objective is to identify the stiffness $k_2$, only the positive branches of the backbone curves are considered. These curves are shown in Fig. 12, where blue lines with up-triangle markers represent the experimental backbone curves for three cases, respectively. The effective stiffness can also be approximated using Eq. (15). The values of $k_2$ were then obtained by substituting the effective stiffness from Eq. (15), together with the experimentally measured positive peak displacements, resonant frequencies, and gaps, into Eq. (14). The corresponding identified backbone curves are shown in Fig. 12 as pink lines with down-triangle markers. The corresponding identified stiffness values of $k_2$ for three cases are 721 N/m, 908.79 N/m and 1944.14 N/m, respectively. These identified values correspond to relative errors of 5.58%, 10.26%, and 5.54%, respectively, compared with the theoretical stiffness values. The results suggest that the backbone-curve-based procedure can provide a useful complementary estimate of the impact stiffness. Furthermore, as illustrated in Fig. 12, the fitted curves follow the general trend of the experimentally extracted peak-response loci. The deviations between the backbone-curve-based stiffness estimates and the theoretical values may be associated with several factors, including measurement uncertainty, the finite frequency resolution of the stepped-sine tests, and the approximate effective-stiffness relation used in Eq. (15). A smaller frequency increment would allow the peak-response points to be located more precisely and may further improve the backbone-curve-based estimate. Nevertheless, with the frequency increment of 0.2 Hz used in the present experiments, the identified stiffness values remain close to the theoretical beam-stiffness estimates. Therefore, the present results are used to demonstrate the practical feasibility of the backbone-curve-based stiffness estimation framework under the adopted experimental protocol.

15 $$\begin{aligned} k_\text {eq} = m_1 {\omega }^2 =m_1 (2\pi f)^2 \end{aligned}$$

where *f* is the resonant frequency.

**Fig. 13:**
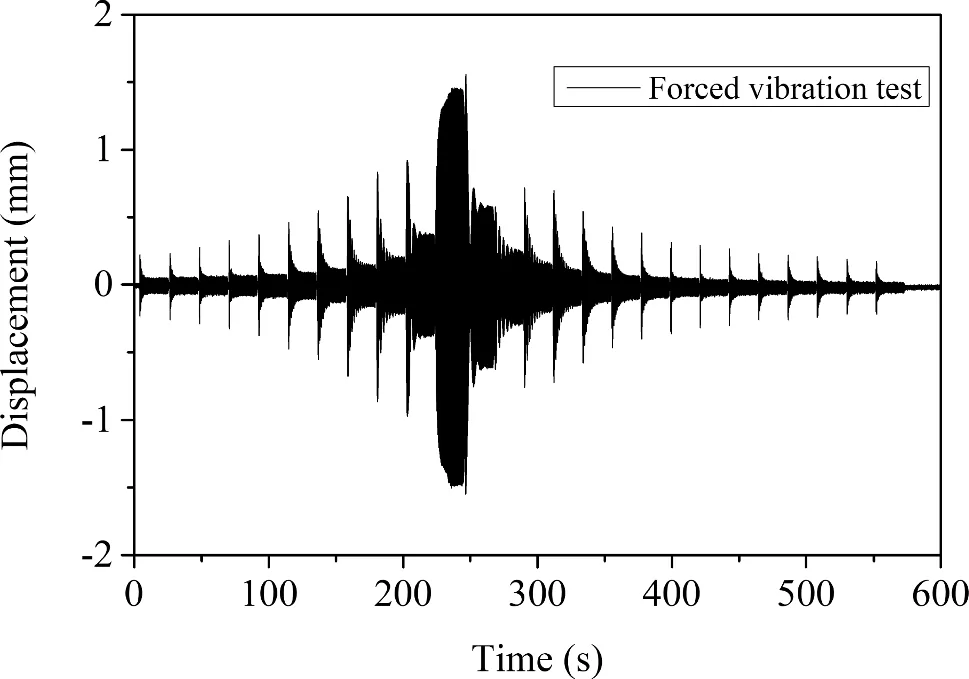
Forced vibration test under the condition that the excitation acceleration is 0.01 g and the frequency was varied from 10 to 15 Hz in increments of 0.2 Hz, with a resonant frequency observed at 12 Hz

Based on Eq. (15), stiffness $k_1$ of the soft spring can also be identified by conducting a forced vibration test. The external excitation acceleration was set to 0.01 g, and the excitation frequency was varied from 10 to 15 Hz in increments of 0.2 Hz. The corresponding time-domain response is shown in Fig. 13. As observed in the figure, the system response remains symmetric throughout the test, indicating that no impacts occurred. The resonant frequency was identified as 12 Hz, and using Eq. (15), the stiffness $k_1$ is estimated as 795.88 N/m, which matches the theoretical value of 800 N/m well.

### Analysis of the influence of the experimental gap on the identified results

As can be seen in Eq. (14), the effective stiffness is also influenced by the gap, which in turn affects the resonant frequency of the system. A special case of Eq. (14) arises when the gap is zero. Under this condition, Eq. (14) can be simplified to Eq. (16), where the effective stiffness becomes a constant. Consequently, the resonant frequency remains independent of the excitation level.

16 $$\begin{aligned} k_\text {eq} = k_1 + \frac{k_2}{2}. \end{aligned}$$

In this subsection, the effect of the experimental gap on the identified results is investigated. Three scenarios for Case 1 labelled as Case 1-2, Case 1-3, and Case 1-4, each with a theoretical beam stiffness of $k_t = 682.9$ N/m, were examined under different experimental gap conditions. The gaps in the experiments were set to 0 mm, 0.53 mm, and 1.15 mm, respectively. The excitation level ranged from 0.06 to 0.12 g in increments of 0.02 g, and the sweep frequency is defined according to the specific conditions of each scenario.

**Fig. 14:**
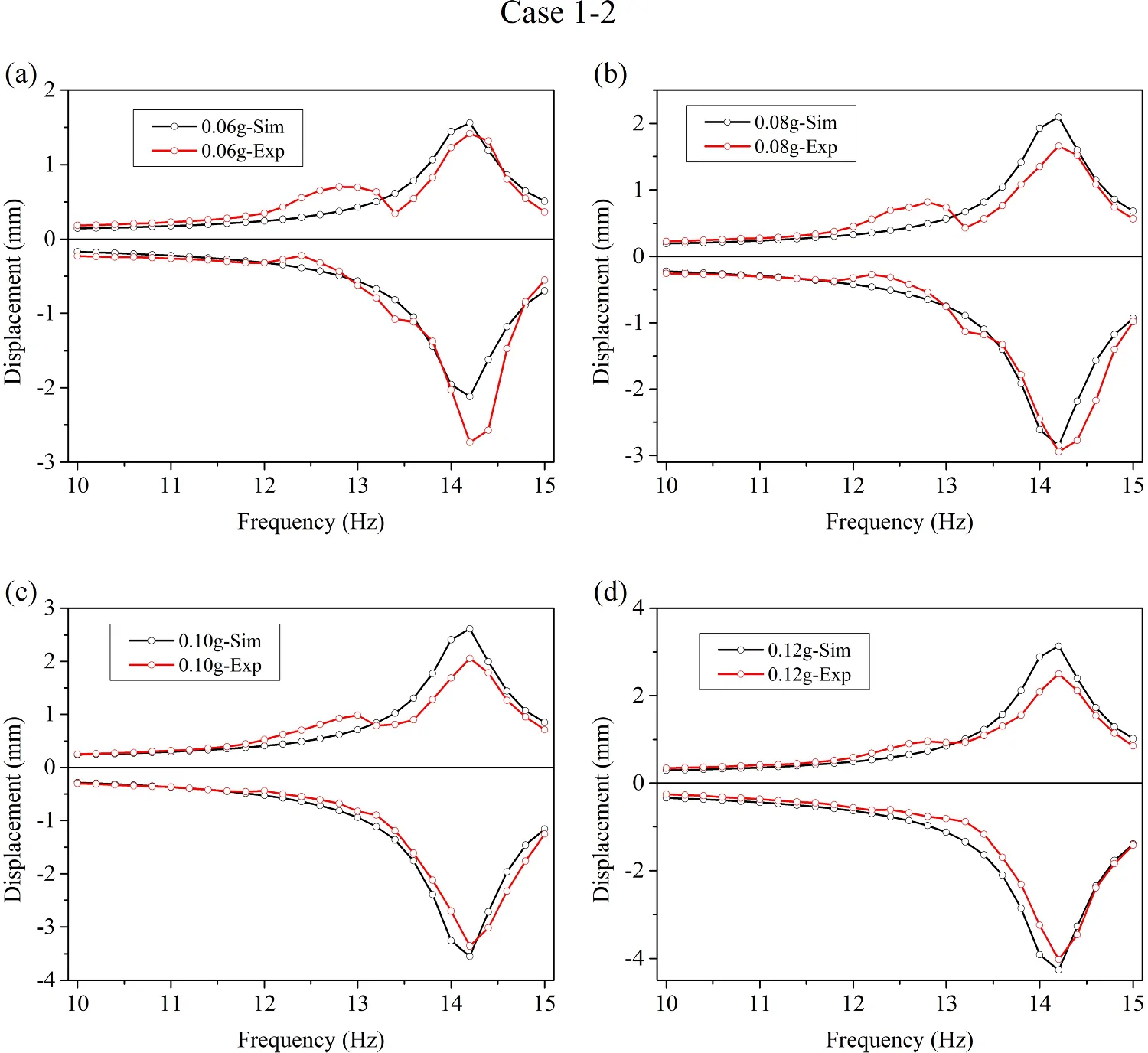
Comparison of the simulated (black solid curves with open-circle markers) and experimental (red solid curves with open-circle markers) positive- and negative-peak displacement–frequency response curves for Case 1-2, with an experimental gap of $g_0 = 0$ mm, at four base-excitation acceleration levels: **a** 0.06 g, **b** 0.08 g, **c** 0.10 g and **d** 0.12 g

**Fig. 15:**
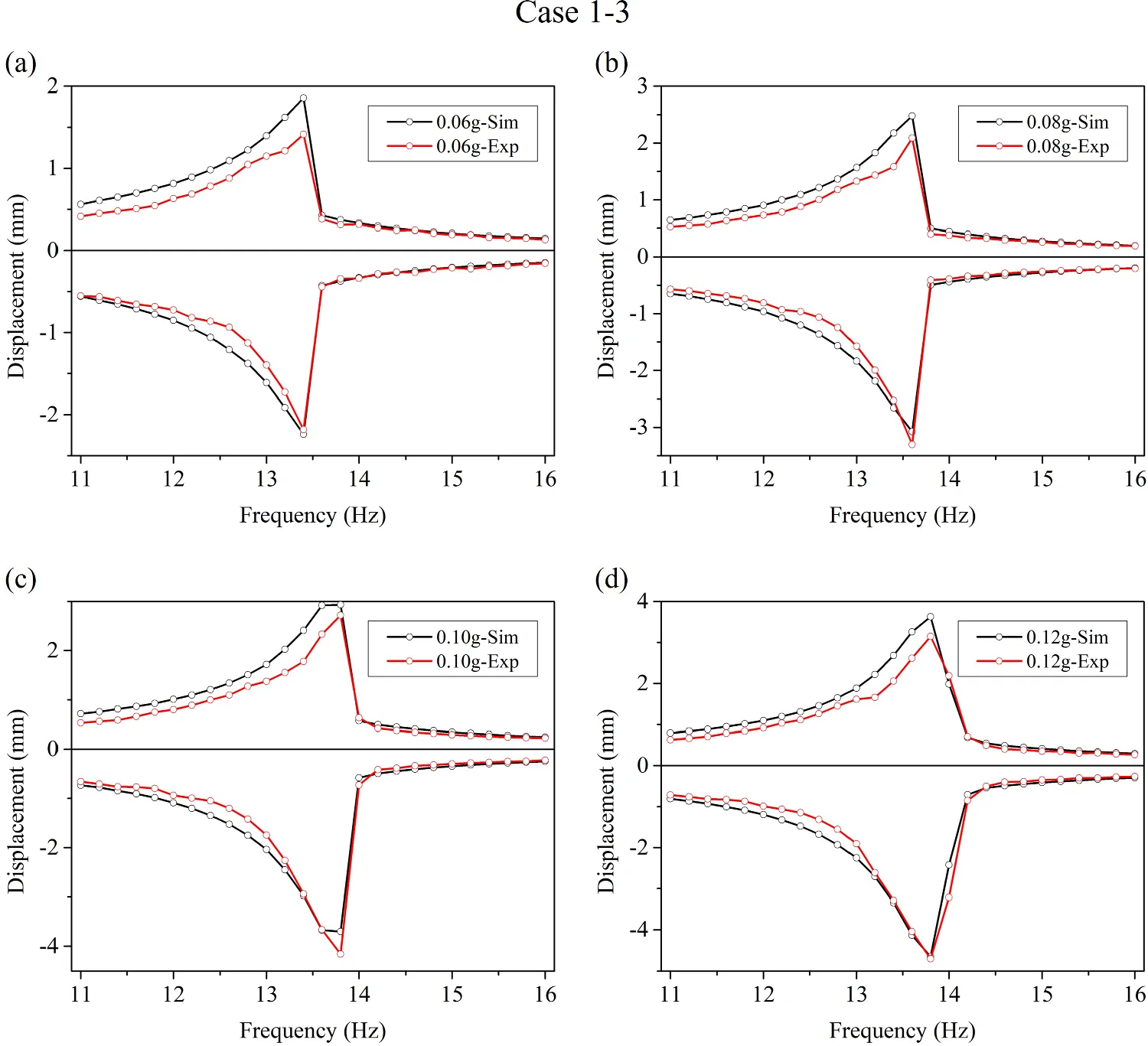
Comparison of the simulated (black solid curves with open-circle markers) and experimental (red solid curves with open-circle markers) positive- and negative-peak displacement–frequency response curves for Case 1-3, with an experimental gap of $g_0 = 0.53$ mm, at four base-excitation acceleration levels: **a** 0.06 g, **b** 0.08 g, **c** 0.10 g and **d** 0.12 g

**Fig. 16:**
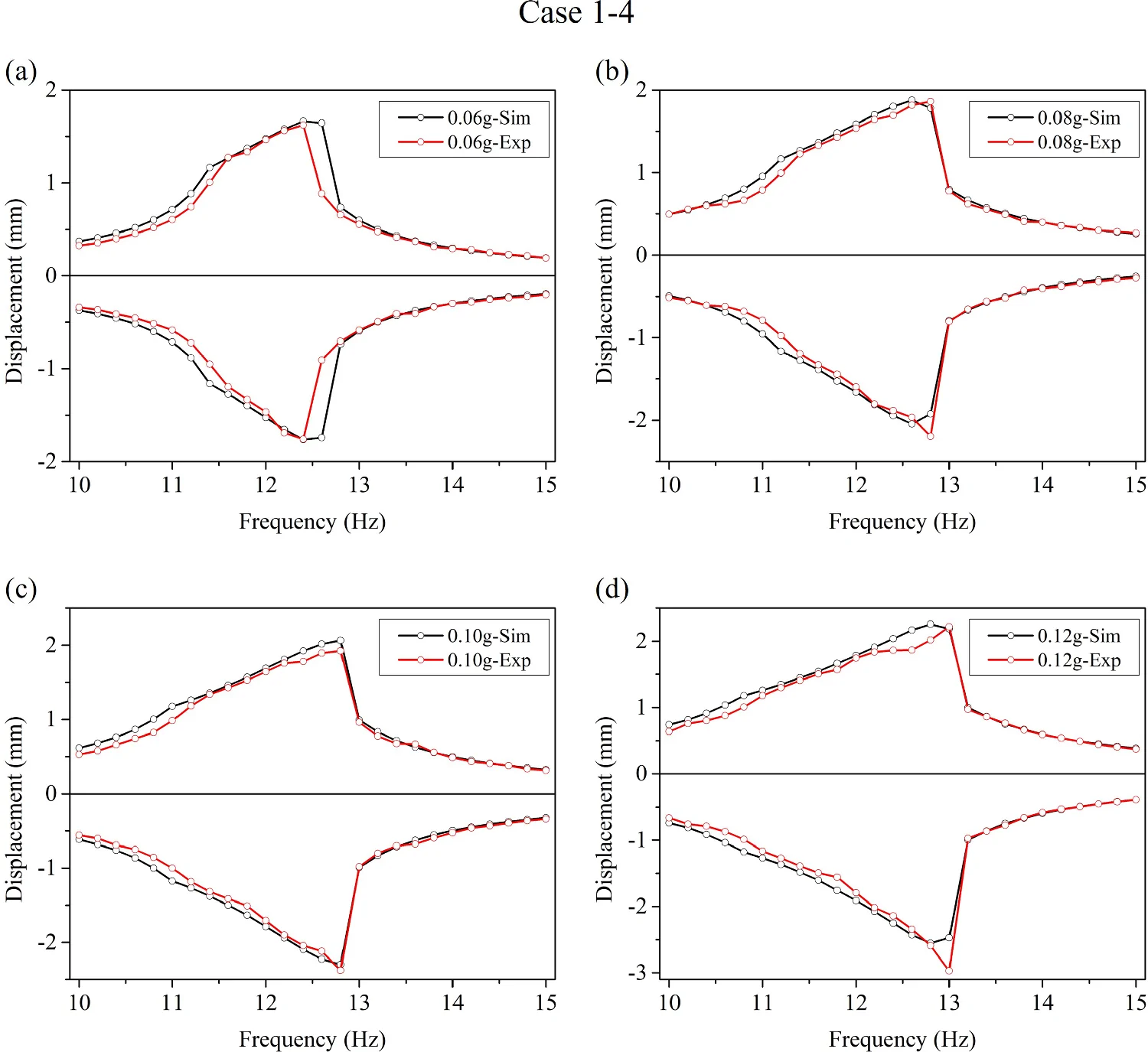
Comparison of the simulated (black solid curves with open-circle markers) and experimental (red solid curves with open-circle markers) positive- and negative-peak displacement–frequency response curves for Case 1-4, with an experimental gap of $g_0 = 1.15$ mm, at four base-excitation acceleration levels: **a** 0.06 g, **b** 0.08 g, **c** 0.10 g and **d** 0.12 g

**Fig. 17:**
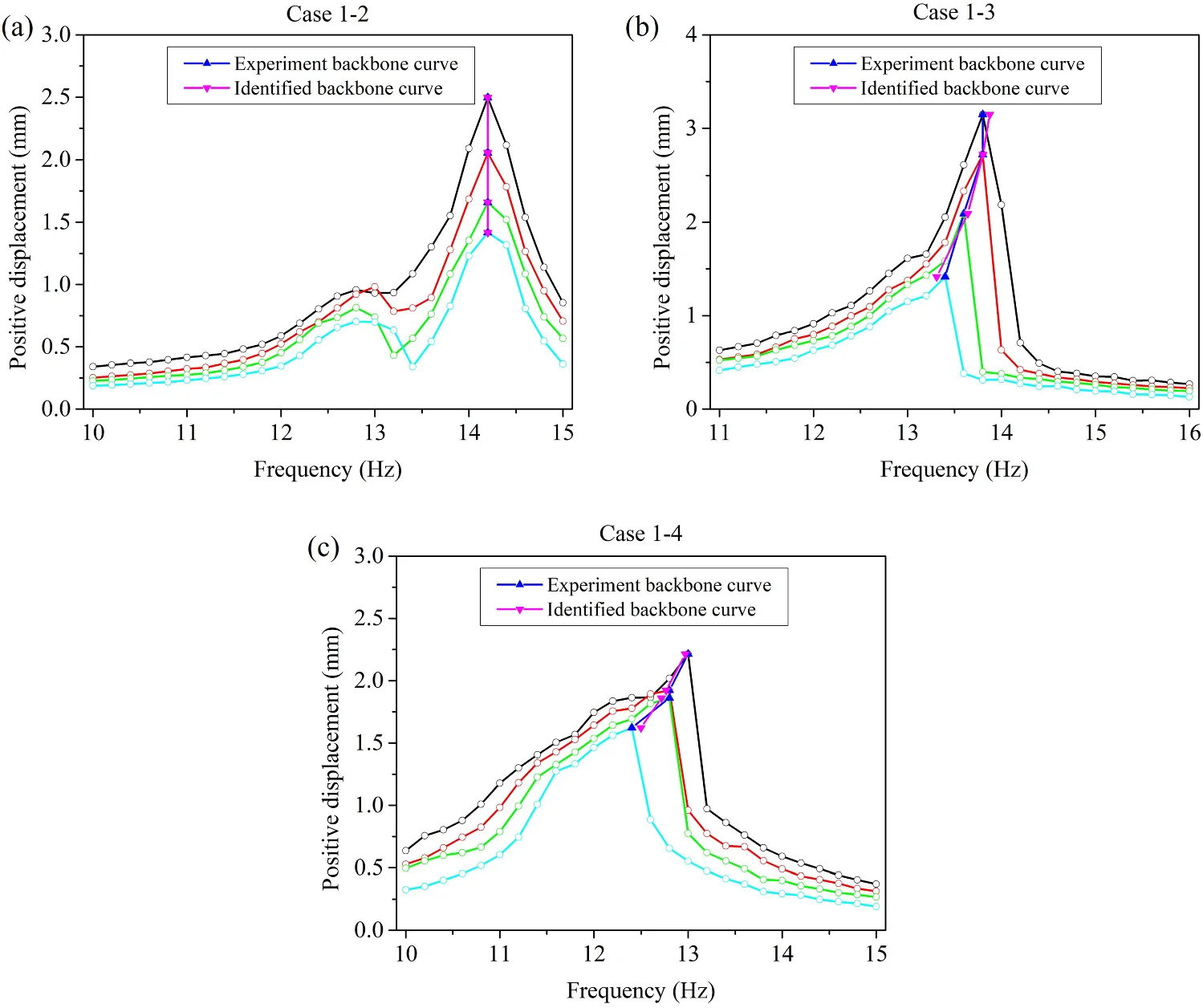
Experimental backbone curves (blue upward-triangle markers) and identified backbone curves (pink downward-triangle markers) for Case 1 under three experimental gap conditions: **a** Case 1-2, $g_0$ = 0 mm; **b** Case 1-3, $g_o$ = 0.53 mm; and **c** Case 1-4, $g_o$ = 1.15 mm

Based on the experimental data, the GA and backbone curve technique are also utilised to identify the stiffness parameter $k_2$. The experimental and simulated displacement–frequency response curves are presented in Figs. 14–16. The experimental and identified backbone curves are shown in Fig. 17. The values of $k_2$ identified using the GA and the backbone-curve method are listed in Table 4.

For Case 1-2, where the gap $g_0=0$ mm, the resonant frequency remains constant at 14.2 Hz across all excitation levels, as shown in Figs. 14 and  17a, which is consistent with the theoretical prediction in Eq. (16). The stiffness $k_2$ identified using the GA is 702.5458 N/m, which is close to $k_t$, whereas the backbone curve technique yields a lower value of 628.92 N/m. By comparing the experimental and simulated displacement–frequency curves, a small local bump can be observed in the experimental response around 13 Hz, particularly at lower excitation levels. This feature is not captured by the current impact model and is likely associated with weak unmodelled contact dynamics. In Case 1-2, the nominal gap is 0 mm, so the system operates close to continuous or near-continuous contact, making the response more sensitive to small alignment errors, local contact geometry, beam clamping compliance, mass-block tilting, and possible contact–separation transitions. As the excitation amplitude increases, the main vibro-impact response becomes dominant and the local bump becomes less pronounced. The bump is therefore interpreted as a local experimental feature associated with unmodelled contact dynamics, rather than as a change in the main stiffness trend used for parameter identification. Despite the observed differences between the experimental and simulated results, the identified stiffness values remain close to the theoretical estimate, with the corresponding relative errors reported in Table 4.

For Case 1-3 ($g_0 = 0.53$ mm) and Case 1-4 ($g_0 = 1.15$ mm), the resonant frequency increases with the excitation amplitude. For instance, in Case 1-3, the resonant frequency is 13.4 Hz under an excitation of 0.06 g in Fig. 15a, whereas it rises to 13.8 Hz under 0.12 g, as depicted in Fig. 15d. The identified stiffness values for both cases agree with the theoretical value within a 5% margin of error.

**Fig. 18:**
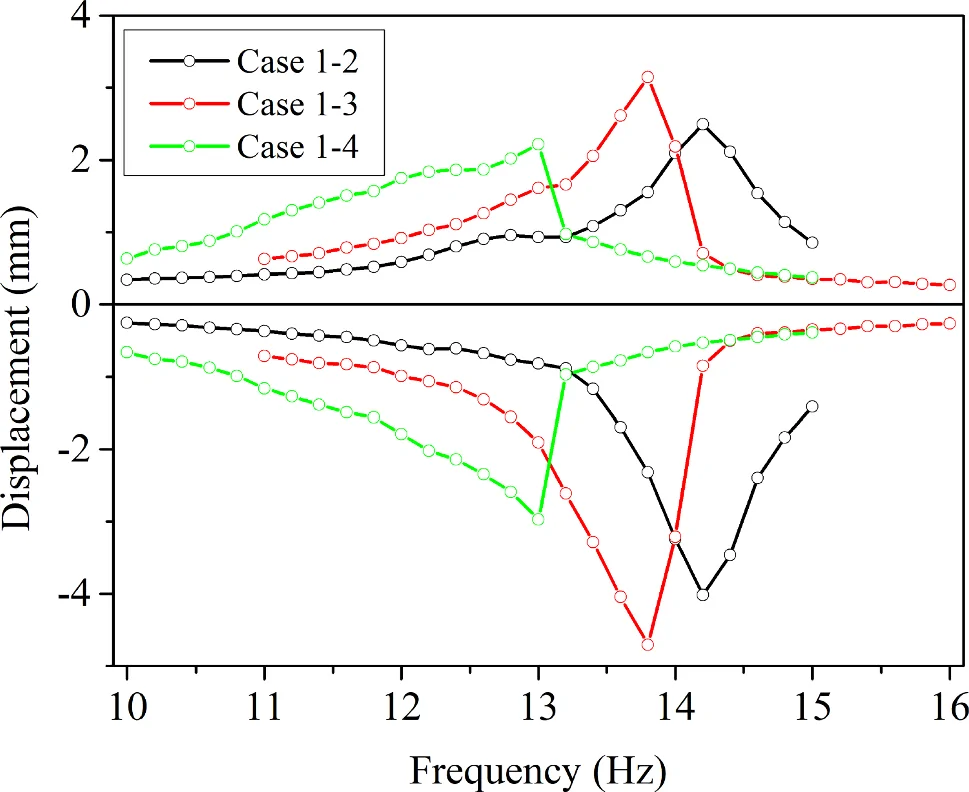
Comparison of the resonant frequencies for Case 1 under a constant external excitation acceleration of 0.12 g and varying experimental gaps, which are 0 mm (black curve), 0.53 mm (red curve), and 1.15 mm (green curve), demonstrating that smaller gaps result in higher resonant frequencies

Comparison of the positive displacement–frequency curves at an excitation acceleration of 0.12 g in Fig. 18 shows that the resonant frequency decreases as the experimental gap increases. For example, the resonant frequency for Case 1-4 is 13 Hz, whereas it is 14.2 Hz for Case 1-2.

Overall, despite variations in the experimental setup, both the GA and the backbone curve technique provide consistent stiffness estimates under the tested gap conditions. The results show that each method is capable of producing accurate estimates within an error margin of approximately 10%, supporting their applicability to the tested forward-sweep response data.

### Repeatability assessment of parameter identification

To assess the repeatability of the identified stiffness parameter, a series of repeated experiments were conducted in this study. While parameter identification techniques such as the GA and the backbone curve method have demonstrated good performance under the various conditions examined in the preceding subsections, it is essential to verify the consistency of their results under repeated experimental runs. Therefore, in this subsection, identical test setups corresponding to Case 1-1, Case 2, and Case 3 were used across multiple trials to evaluate the robustness of the identification process. The variation in identified parameters is analysed to assess the influence of measurement noise, experimental uncertainties, and potential non-repeatability in system behaviour. This repeatability assessment provides additional information on the consistency of the proposed identification methods.

**Fig. 19:**
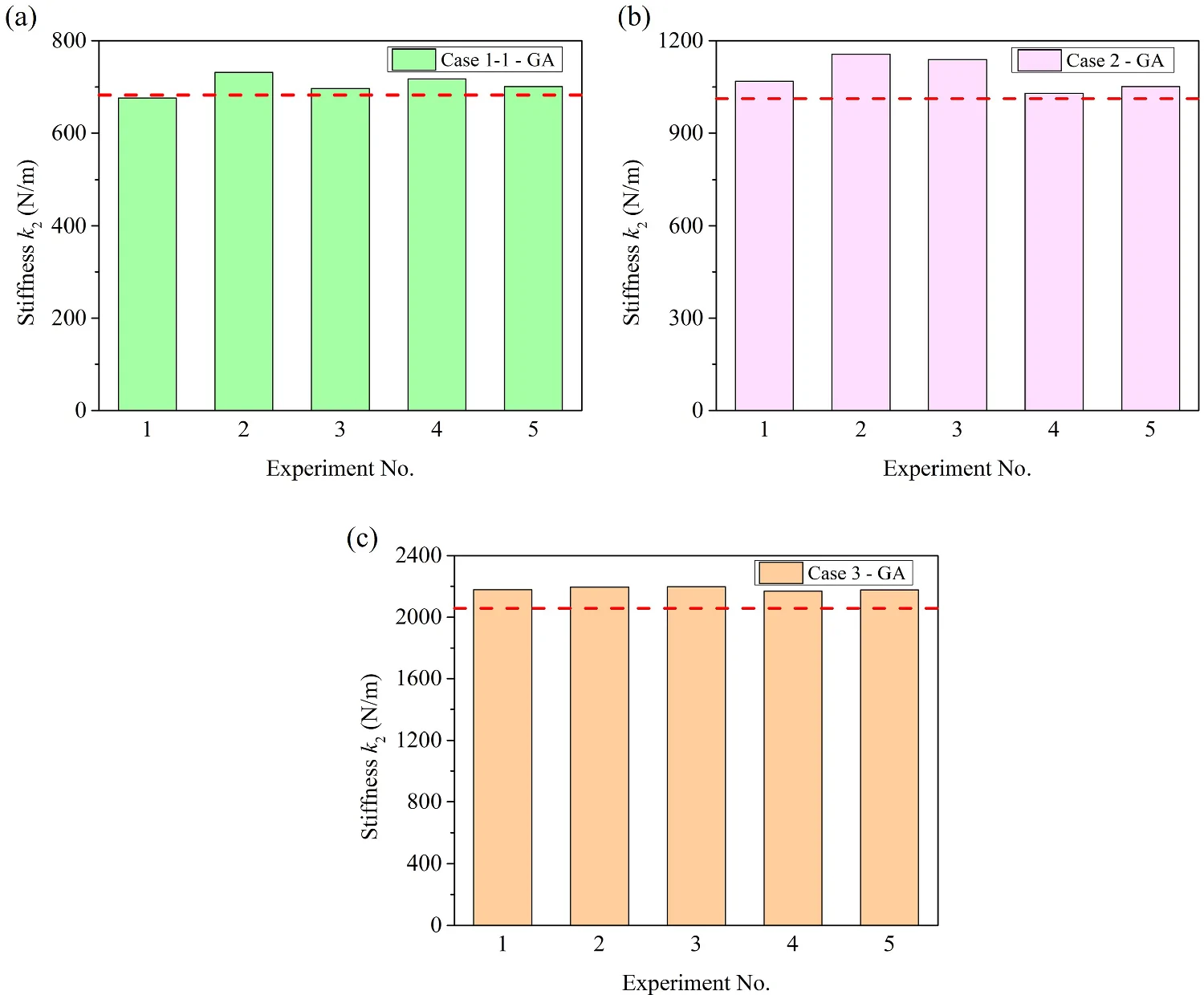
Experimental identification results of stiffness $k_2$ represented as column plots for five trials conducted under the same experimental setup using the GA for **a** Case 1-1, **b** Case 2 and **c** Case 3, where the red dashed line indicates the theoretical reference value presented in Table 5

**Fig. 20:**
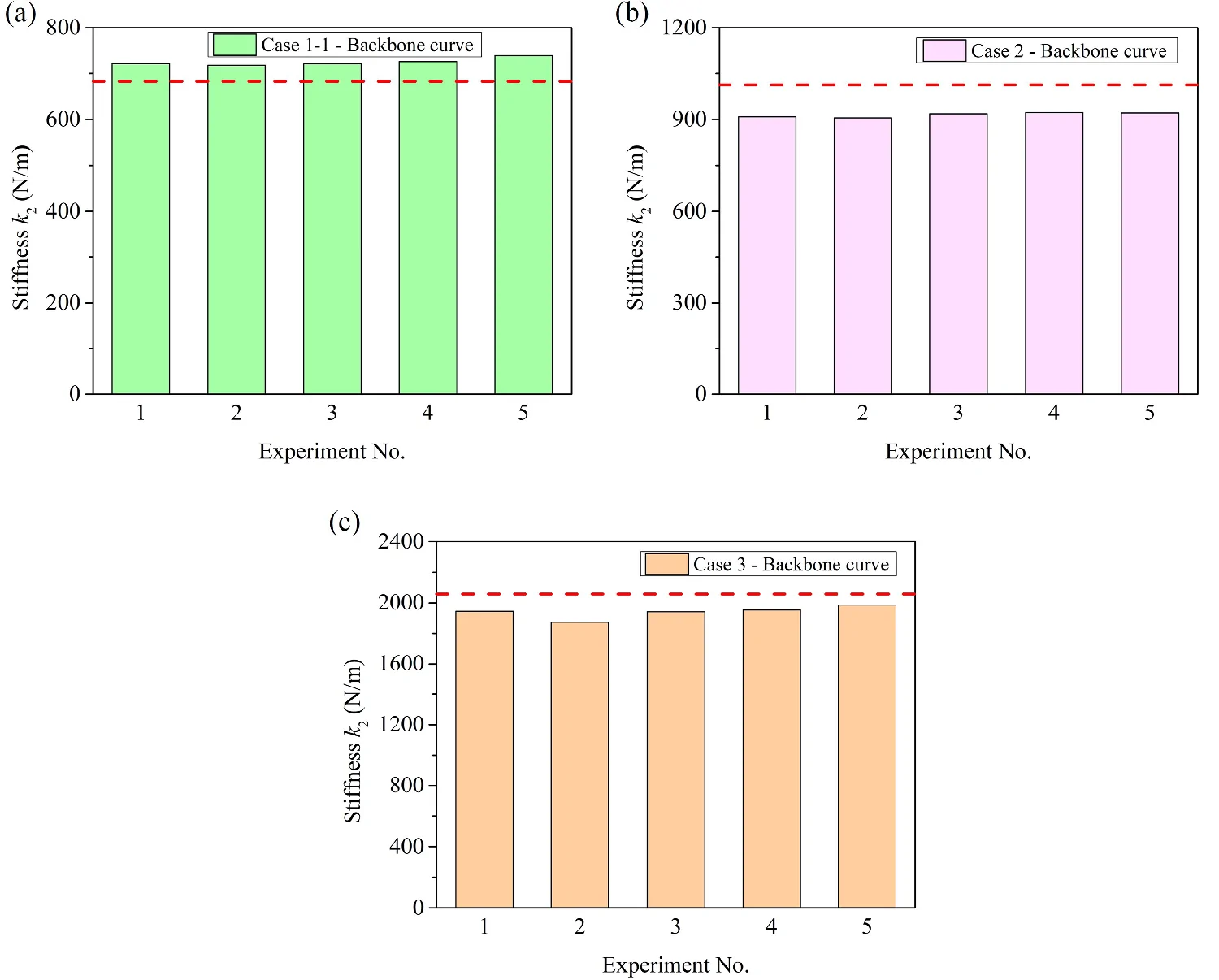
Experimental identification results of stiffness $k_2$ represented as column plots for five trials conducted under the same experimental setup using the backbone-curve method for **a** Case 1-1, **b** Case 2 and **c** Case 3, where the red dashed line indicates the theoretical reference value presented in Table 6

In this study, five repeated experiments were conducted for each case. Tables 5 and  6 summarise the identified stiffness values and corresponding error percentages for each method across five repeated trials per case, while Figs. 19 and 20 provide the visual comparison of results relative to the theoretical reference values, which are indicated by red dashed lines.

**Table 4:** Identified values of $k_2$ obtained from the GA and fitting the backbone curves for Case 1 under three different experimental gaps

Case	Gap $g_0$ (mm)	$k_2$ - GA (N/m)	$k_2$ - Backbone curve (N/m)	$k_t$ (N/m)	$E_{k_2}$-GA	$E_{k_2}$-Backbone curve
1-2	0	702.5458	628.92	682.9	2.88%	7.90%
1-3	0.53	709.0688	671.45	682.9	3.83%	1.68%
1-4	1.15	699.6929	701.36	682.9	2.46%	2.70%

For the GA, the identified stiffness values exhibit good repeatability across all cases. In Case 1-1, with a theoretical stiffness of 682.9 N/m, the identified values vary within a 7.2% range, with most errors below 5%, indicating a high level of consistency. Case 2 shows a slightly wider spread, with errors ranging from 1.59% to 14.20%. This larger variation may be associated with the contact regime observed for this configuration. Compared with Cases 1-1 and 3, the responses in Case 2 show a more noticeable difference between the simulated and experimental negative displacement branches, indicating that the identified stiffness is more affected by small changes in gap setting, contact timing, damping, or mass-block alignment. In Case 3, where the theoretical stiffness is 2058.1 N/m, the results are notably stable, with all five trials producing errors below 6.8%.

In contrast, the backbone curve fitting method shows slightly larger and more consistent errors across trials. In Case 1-1, the identified stiffness values range from 717.29 to 739.46 N/m, corresponding to error percentages between 5.04% and 8.28%. Case 2 results are generally lower than the theoretical value, with errors between 8.91% and 10.66%. In Case 3, the method again performs better, yielding errors mostly within 6%, except for one trial with an 8.99% deviation.

**Table 5:** Comparison of the identified values of $k_2$ obtained from the GA with the theoretical values $k_t$, along with the corresponding error percentages for (a) Case 1-1, (b) Case 2 and (c) Case 3

Exp No	Identified $k_2$ (N/m)	$k_t$ (N/m)	$E_{k_2}$ (%)
(a) *Case 1-1*			
1	676.77	682.9	0.90
2	732.02	682.9	7.19
3	696.63	682.9	2.01
4	717.48	682.9	5.06
5	700.89	682.9	2.63
(b) *Case 2*			
1	1068.51	1012.7	5.51
2	1156.71	1012.7	14.20
3	1139.70	1012.7	12.54
4	1028.81	1012.7	1.59
5	1051.20	1012.7	3.80
(c) *Case 3*			
1	2177.68	2058.1	5.81
2	2194.11	2058.1	6.61
3	2197	2058.1	6.75
4	2167.3	2058.1	5.31
5	2176.67	2058.1	5.76

**Table 6:** Comparison of the identified values of $k_2$ obtained from fitting the backbone curves with the theoretical values $k_t$, along with the corresponding error percentages for (a) Case 1-1, (b) Case 2 and (c) Case 3

Exp No	Identified $k_2$ (N/m)	$k_t$ (N/m)	$E_{k_2}$ (%)
(a) *Case 1-1*			
1	721	682.9	5.58
2	717.29	682.9	5.04
3	720.95	682.9	5.57
4	726.2	682.9	6.34
5	739.46	682.9	8.28
(b) *Case 2*			
1	908.79	1012.7	10.26
2	904.73	1012.7	10.66
3	917.85	1012.7	9.37
4	922.44	1012.7	8.91
5	920.97	1012.7	9.06
(c) *Case 3*			
1	1944.14	2058.1	5.54
2	1872.99	2058.1	8.99
3	1940.23	2058.1	5.73
4	1951.39	2058.1	5.18
5	1983.63	2058.1	3.62

The bar plots in Figs. 19 and 20 confirm these trends visually. The GA-based results are more tightly clustered around the theoretical values, particularly in Cases 1-1 and 3. The backbone curve results are slightly dispersed, but the identified stiffness values remain close to the theoretical values, as quantified by the corresponding error percentages. These results indicate that the method provides repeatable stiffness estimates for the tested cases.

The slightly larger errors obtained from the backbone-curve method are consistent with the nature of this approach. The method compresses each frequency-response curve into a limited number of resonant points and estimates the effective stiffness from these points. As a result, the identified stiffness is strongly influenced by the frequency resolution, the accuracy of peak detection, and the approximation involved in converting resonant frequency into equivalent stiffness. The GA-based method, by contrast, uses the full displacement–frequency response curves and separately accounts for the positive and negative displacement branches, which provides more information for the optimisation and improves the consistency of the identified parameters.

In summary, both the GA and backbone-curve-based methods show repeatable stiffness estimates across the tested stiffness cases under the same experimental protocol. The GA demonstrates slightly better consistency and accuracy, particularly in low- and high-stiffness scenarios. These findings support the consistency of both techniques under the tested experimental conditions and support their application in practical experimental settings for nonlinear system identification.

## Conclusions

This study presented a comprehensive investigation into the dynamic behaviour and parameter identification of an impact oscillator incorporating a one-sided elastic constraint. A custom-designed experimental rig was developed to provide controlled forward stepped-sine excitation, enabling systematic exploration of nonlinear vibro-impact responses. A piecewise-defined mathematical model was formulated to describe the unilateral contact dynamics, with non-negativity conditions imposed on the contact force to ensure physical consistency.

Two complementary identification strategies, a GA-based optimisation method and a backbone curve fitting approach derived from harmonic balance principles, were employed to estimate the secondary stiffness parameter $k_2$. The relative errors between the identified stiffness values and the theoretical beam-stiffness estimates were reported for each case, allowing a more direct comparison between the GA-based and backbone-curve-based procedures.

The influence of the impact gap on the system dynamics was analysed, revealing a clear dependency of the resonant frequency on gap size: larger gaps reduce the effective system stiffness, leading to lower resonant frequencies. The repeatability of both identification methods was assessed through five repeated experimental trials. The identified stiffness values were compared with the corresponding theoretical beam-stiffness estimates using the relative errors reported in the tables. The GA-based method generally showed more consistent stiffness estimates across the tested cases, while the backbone-curve-based method provided a useful complementary estimate of the impact stiffness. A full robustness assessment involving uncertainty propagation will be considered in future studies.

Overall, the findings indicate that the proposed framework can provide consistent stiffness estimates for the tested one-sided impact oscillator under the present experimental conditions. The methodology developed here provides a reliable basis for future extensions to more complex structures or biomedical diagnostic applications, including vibro-impact sensing for bowel cancer detection [61]. Moreover, full characterisation of hysteresis ranges, branch transitions, unstable branches, and coexisting response regimes will be pursued in future work using upward and downward sweep tests.

## Data Availability

The numerical and experimental data sets generated and analysed during the current study are available from the corresponding author on reasonable request.

## Derivation of the effective stiffness of the impact system with bilinear stiffness

The mathematical model of the impact oscillator analysed in this work is described by the following equation of motion:

$$\begin{aligned}&m_1\ddot{y}+c_{1}\dot{y}+c_{2}\dot{y}\cdot H(y-g_0)+k_{1}y \\&\qquad +k_{2}(y-g_0)\cdot H(y-g_0)=F\sin (\omega t), \end{aligned}$$

where H(·) is the Heaviside step function, and $g_0$ is the gap between the mass block and elastic beam. Based on this, the restoring force can be defined as

$$ f_s(y) = {\left\{ \begin{array}{ll} k_1y, &  \text {if } \ \ y < g_0, \\ k_1y + k_2(y-g_0), &  \text {if } \ \ y \ge g_0. \end{array}\right. } $$

Assuming a trial solution of motion

$$ y(t) = Y \sin (\omega t) $$

When $y(t) = g_0$, we have:

$$ g_0 = Y \sin (\omega t) \quad \Rightarrow \quad \omega t = \sin ^{-1} \left( \frac{g_0}{Y} \right) $$

Therefore, the phase angle $\theta _c$ at which the displacement *y* first reaches the gap $g_0$ can be defined as:

$$ \theta _c = \sin ^{-1} \left( \frac{g_0}{Y} \right) $$

This indicates that within the interval $-\theta _c$ to $+\theta _c$, the motion stays within the initial linear region. For angles $\theta _c \le \theta \le \pi - \theta _c$, the response enters the second stiffness region. Although both regions exhibit linear behaviour individually, the overall system is nonlinear due to the piecewise nature of the stiffness definition.

The effective stiffness of the system is defined as:

$$ k_{\text {eq}} = \frac{1}{\pi Y^2} \int _0^\pi f_s(Y \sin \theta ) \cdot Y \sin \theta \, \text {d}\theta $$

Due to the presence of the gap $g_0$, the system is divided into two regions:

$$ 0 \le \theta < \theta _c \quad \text {(initial linear stiffness region, only } k_1 \text { is active)} $$

$$\begin{aligned}&\theta _c \le \theta < \pi - \theta _c \\&\quad \quad \text {(nonlinear stiffness region, both } k_1 \text { and } k_2 \text { are active)} \end{aligned}$$

Therefore, the stiffness $k_1$ remains active throughout the motion. In the nonlinear region, the restoring force function $f_s(Y \sin \theta )$ is given by:

$$ f_s(Y \sin \theta ) = k_1 Y \sin \theta + k_2 (Y \sin \theta - g_0) $$

To simplify the expression, the equation of the effective stiffness can be rewritten as:

$$ k_{\text {eq}} = k_1 + \frac{k_2}{\pi } \int _{\theta _c}^{\pi - \theta _c} \sin \theta \left( \sin \theta - \frac{g_0}{Y} \right) \text {d}\theta $$

This formulation highlights the separate contributions of the linear and nonlinear stiffness components.

To evaluate the integral in the equation, we define:

$$ I = \int _{\theta _c}^{\pi - \theta _c} \sin ^2 \theta \, \text {d}\theta - \frac{g_0}{Y} \int _{\theta _c}^{\pi - \theta _c} \sin \theta \, \text {d}\theta $$

Using standard trigonometric integrals, we can get

$$ \int _{\theta _c}^{\pi - \theta _c} \sin ^2 \theta \, \text {d}\theta = \frac{1}{2} \left( \pi - 2\theta _c + \sin (2\theta _c) \right) , $$

$$ \int _{\theta _c}^{\pi - \theta _c} \sin \theta \, \text {d}\theta = 2\cos \theta _c $$

Then, we get the final result of the integral as follows

$$ I = \frac{\pi - 2\theta _c}{2} + \frac{\sin 2\theta _c}{2} - \frac{g_0}{Y} \cdot 2\cos \theta _c $$

Substituting the computed integral into the equation of the effective stiffness, we obtain the final expression for the effective stiffness:

$$ k_{\text {eq}} = k_1 + \frac{k_2}{2\pi } \left[ \pi - 2 \theta _c + \sin (2\theta _c) - \frac{4 g_0}{Y} \cos \theta _c \right] $$

This expression quantifies the equivalent stiffness of the impact oscillator with bilinear stiffness.

## References

[1] Pavlovskaia, E., Wiercigroch, M., Woo, K.-C., Rodger, A.A.: Modelling of ground moling dynamics by an impact oscillator with a frictional slider. Meccanica **38**, 85–97 (2003)

[2] Pavlovskaia, E., Wiercigroch, M.: Periodic solution finder for an impact oscillator with a drift. J. Sound Vib. **267**(4), 893–911 (2003)

[3] Qiu, P.Z., Tan, Y., Thompson, O., Cobley, B., Nanayakkara, T.: Soft tissue characterisation using a novel robotic medical percussion device with acoustic analysis and neural networks. IEEE Robotics and Automation Letters **7**(4), 11314–11321 (2022)

[4] Ema, S., Marui, E.: Damping characteristics of an impact damper and its application. Int. J. Mach. Tools Manuf **36**(3), 293–306 (1996)

[5] Blazejczyk-Okolewska, B.: Analysis of an impact damper of vibrations. Chaos, Solitons & Fractals **12**(11), 1983–1988 (2001)

[6] Cheng, C., Wang, J.: Free vibration analysis of a resilient impact damper. Int. J. Mech. Sci. **45**(4), 589–604 (2003)

[7] Serdukova, L., Kuske, R., Yurchenko, D.: Post-grazing dynamics of a vibro-impacting energy generator. J. Sound Vib. **492**, 115811 (2021)

[8] Costa, D., Kuske, R., Yurchenko, D.: Qualitative changes in bifurcation structure for soft vs hard impact models of a vibro-impact energy harvester, Chaos: An Interdisciplinary Journal of Nonlinear Science, **32**, 103120 (2022) 10.1063/5.0101050 36319279

[9] Guo, B., Liu, Y., Birler, R., Prasad, S.: Self-propelled capsule endoscopy for small-bowel examination: proof-of-concept and model verification. Int. J. Mech. Sci. **174**, 105506 (2020)

[10] Liu, Y., Pavlovskaia, E., Wiercigroch, M.: Experimental verification of the vibro-impact capsule model. Nonlinear Dyn. **83**, 1029–1041 (2016)

[11] Páez Chávez, J., Liu, Y., Pavlovskaia, E., Wiercigroch, M.: Path-following analysis of the dynamical response of a piecewise-linear capsule system. Commun. Nonlinear Sci. Numer. Simul. **37**, 102–114 (2016)

[12] Liu, Y., Páez Chávez, J., Zhang, J., Tian, J., Guo, B., Prasad, S.: The vibro-impact capsule system in millimetre scale: numerical optimisation and experimental verification. Meccanica **55**, 1885–1902 (2020)

[13] Nordmark, A.B., Piiroinen, P.T.: Simulation and stability analysis of impacting systems with complete chattering. Nonlinear Dyn. **58**(1), 85–106 (2009)

[14] Liu, Y., Páez Chávez, J., Guo, B., Birler, R.: Bifurcation analysis of a vibro-impact experimental rig with two-sided constraint. Meccanica **55**(12), 2505–2521 (2020)

[15] Yin, S., Shen, Y., Wen, G., Xu, H.: Analytical determination for degenerate grazing bifurcation points in the single-degree-of-freedom impact oscillator. Nonlinear Dyn. **90**(1), 443–456 (2017)

[16] Yin, S., Wen, G., Wu, X.: Suppression of grazing-induced instability in single degree-of-freedom impact oscillators. Appl. Math. Mech. **40**(1), 97–110 (2019)

[17] Rosenfeld, J.A., Russo, B.P., Kamalapurkar, R., Johnson, T.T.: The occupation kernel method for nonlinear system identification. SIAM J. Control. Optim. **62**(3), 1643–1668 (2024)

[18] Kim, T.: Kernel regression-based state-space estimation of continuous-time dynamic systems from noisy sampled state data. IEEE Trans. Autom. Control **69**(7), 4757–4764 (2024)

[19] Alferink, D., Fey, R., Van De Wouw, N., Heertjes, M.: Hysteresis in motion control systems: A frequency domain approach, in 2024 European Control Conference (ECC), 2825–2830, IEEE, (2024)

[20] Zhao, Q., Wang, F., Wang, W., Zhang, T., Wu, H., Ning, W.: Research on intrusion detection model based on improved mlp algorithm. Sci. Rep. **15**(1), 5159 (2025) 39939428 10.1038/s41598-025-89798-0 PMC11821824

[21] Yu, R., Wang, R.: Learning dynamical systems from data: An introduction to physics-guided deep learning. Proc. Natl. Acad. Sci. **121**(27), e2311808121 (2024) 38913886 10.1073/pnas.2311808121 PMC11228478

[22] Giacomuzzo, G., Carli, R., Romeres, D., Dalla Libera, A.: A black-box physics-informed estimator based on gaussian process regression for robot inverse dynamics identification, IEEE Transactions on Robotics, 40, 4820–4836 (2024)

[23] Wang, Y., Qian, H., Ma, Y., Liu, Q., Zhu, R., Jiang, D.: Reliable sparse identification of nonlinear continuous structural dynamics via subspace-based feature transformation and bayesian priors, Nonlinear Dynamics, 113, 13081–13096 (2025)

[24] Wang, Y., Qian, H., Liu, Q., Ma, Y., Jiang, D.: Hierarchical bayesian model for identifying clearance-type nonlinear system. Mech. Syst. Signal Process. **235**, 112891 (2025)

[25] Zhao, S., Cheng, C., Lin, M., Peng, Z.: Physics-informed deep sparse regression network for nonlinear dynamical system identification. J. Sound Vib. **595**, 118796 (2025)

[26] Liang, J., Zhao, Q., Zhao, W.: Nonlinear system identification of air turbine rocket engine based on polynomial nonlinear state space model. Nonlinear Dyn. **113**(14), 17637–17660 (2025)

[27] Ghorbani, E., Dollon, Q., Gosselin, F.P.: Physics-aware tuning of the unscented kalman filter: statistical framework for solving inverse problems involving nonlinear dynamical systems and missing data. Nonlinear Dyn. **113**(5), 4301–4323 (2025)

[28] Amari, S.-I.: Backpropagation and stochastic gradient descent method. Neurocomputing **5**(4–5), 185–196 (1993)

[29] Hochreiter, S., Younger, A.S., Conwell, P.R.: Learning to learn using gradient descent, in International conference on artificial neural networks, 87–94, Springer, (2001)

[30] Elnady, S.M., El-Beltagy, M., Radwan, A.G., Fouda, M.E.: A comprehensive survey of fractional gradient descent methods and their convergence analysis. Chaos, Solitons & Fractals **194**, 116154 (2025)

[31] Dennis, J.E., Jr., Moré, J.J.: Quasi-newton methods, motivation and theory. SIAM Rev. **19**(1), 46–89 (1977)

[32] Broyden, C.G.: Quasi-newton methods and their application to function minimisation. Math. Comput. **21**(99), 368–381 (1967)

[33] Zhang, J., Xu, C.: Properties and numerical performance of quasi-newton methods with modified quasi-newton equations. J. Comput. Appl. Math. **137**(2), 269–278 (2001)

[34] Özdamar, L., Demirhan, M.: Experiments with new stochastic global optimization search techniques. Computers & Operations Research **27**(9), 841–865 (2000)

[35] Zabinsky, Z.B.: Stochastic methods for practical global optimization. J. Global Optim. **13**(4), 433–444 (1998)

[36] Raphael, B., Smith, I.F.: A direct stochastic algorithm for global search. Appl. Math. Comput. **146**(2–3), 729–758 (2003)

[37] Foster, J.A.: Evolutionary computation. Nat. Rev. Genet. **2**(6), 428–436 (2001) 11389459 10.1038/35076523

[38] Back, T., Schwefel, H.-P.: “Evolutionary computation: An overview,” in Proceedings of IEEE International Conference on Evolutionary Computation, 20–29, IEEE, (1996)

[39] Kannan, S., Slochanal, S.M.R., Padhy, N.P.: Application and comparison of metaheuristic techniques to generation expansion planning problem. IEEE Trans. Power Syst. **20**(1), 466–475 (2005)

[40] Abd Elaziz, M., Elsheikh, A.H., Oliva, D., Abualigah, L., Lu, S., Ewees, A.A.: Advanced metaheuristic techniques for mechanical design problems. Archives of Computational Methods in Engineering **29**(1), 695–716 (2022)

[41] Slowik, A., Kwasnicka, H.: Nature inspired methods and their industry applications-swarm intelligence algorithms. IEEE Trans. Industr. Inf. **14**(3), 1004–1015 (2017)

[42] Blum, C.: Ant colony optimization: Introduction and recent trends. Phys. Life Rev. **2**(4), 353–373 (2005)

[43] Kao, C.-C., Fung, R.-F.: Using the modified pso method to identify a scott-russell mechanism actuated by a piezoelectric element. Mech. Syst. Signal Process. **23**(5), 1652–1661 (2009)

[44] Subudhi, B., Jena, D.: A differential evolution based neural network approach to nonlinear system identification. Appl. Soft Comput. **11**(1), 861–871 (2011)

[45] Kramer, O.: Genetic algorithms essentials. Springer, (2017)

[46] Yao, L., Sethares, W.A.: Nonlinear parameter estimation via the genetic algorithm. IEEE Trans. Signal Process. **42**(4), 927–935 (1994)

[47] Kapanoglu, M., Koc, I., Erdogmus, S.: Genetic algorithms in parameter estimation for nonlinear regression models: an experimental approach. J. Stat. Comput. Simul. **77**(10), 851–867 (2007)

[48] Kerschen, G., Worden, K., Vakakis, A.F., Golinval, J.-C.: Past, present and future of nonlinear system identification in structural dynamics. Mech. Syst. Signal Process. **20**(3), 505–592 (2006)

[49] Kerschen, G., Peeters, M., Golinval, J.-C., Vakakis, A.F.: Nonlinear normal modes, part i: A useful framework for the structural dynamicist. Mech. Syst. Signal Process. **23**(1), 170–194 (2009)

[50] Londoño, J.M., Neild, S.A., Cooper, J.E.: Identification of backbone curves of nonlinear systems from resonance decay responses. J. Sound Vib. **348**, 224–238 (2015)

[51] Londoño, J.M., Cooper, J.E., Neild, S.A.: Identification of systems containing nonlinear stiffnesses using backbone curves. Mech. Syst. Signal Process. **84**, 116–135 (2017)

[52] Ondra, V., Sever, I., Schwingshackl, C.: A method for non-parametric identification of non-linear vibration systems with asymmetric restoring forces from a resonant decay response. Mech. Syst. Signal Process. **114**, 239–258 (2019)

[53] Safari, S., Londoño Monsalve, J.M.: Data-driven structural identification of nonlinear assemblies: Asymmetric stiffness and damping nonlinearities. Mech. Syst. Signal Process. **222**, 111745 (2025)

[54] Hill, T., Green, P., Cammarano, A., Neild, S.: Fast bayesian identification of a class of elastic weakly nonlinear systems using backbone curves. J. Sound Vib. **360**, 156–170 (2016)

[55] Karaağaçlı, T., Özgüven, H.N.: Experimental identification of backbone curves of strongly nonlinear systems by using response-controlled stepped-sine testing (rct). Vibration **3**(3), 266–280 (2020)

[56] Tian, B., Yin, S., Liu, Y., Londoño, J.M.: Nonlinear characteristics identification of an impact oscillator with a one-sided elastic constraint. J. Sound Vib. **575**, 118270 (2024)

[57] Tian, B., Yin, S., Páez Chávez, J., Liu, Y.: An optimization approach to establish dynamical equivalence for soft and rigid impact models, Chaos: An Interdisciplinary Journal of Nonlinear Science, **34**, 073106 (2024) 10.1063/5.0209026 38949529

[58] Wang, Z., Tian, J., Feng, K.: Optimal allocation of regional water resources based on simulated annealing particle swarm optimization algorithm. Energy Rep. **8**, 9119–9126 (2022)

[59] Genesio, R., Tesi, A.: Harmonic balance methods for the analysis of chaotic dynamics in nonlinear systems. Automatica **28**(3), 531–548 (1992)

[60] Cheung, Y., Chen, S., Lau, S.: Application of the incremental harmonic balance method to cubic non-linearity systems. J. Sound Vib. **140**(2), 273–286 (1990)

[61] Tian, B., Liu, Y., Londoño, J.M.: Dynamic analysis of a vibrating indentation probe for in-situ bowel cancer diagnosis. Mech. Syst. Signal Process. **235**, 112830 (2025)

